# Single-cell transcriptomics reveals targeted modulation of inflammatory repertoire by SOCE blockers

**DOI:** 10.1016/j.humimm.2026.111687

**Published:** 2026-02-13

**Authors:** Andreas Stephanou, Madhav Mantri, Divya Shankaranarayanan, Carol Li, Mila Lagman, Jenny Xiang, Chendong Pan, Yanjie Sun, Thangamani Muthukumar, Khaled Machaca, Iwijn De Vlaminck, Manikkam Suthanthiran

**Affiliations:** a Nancy E. and Peter C. Meinig School of Biomedical Engineering, Cornell University, Ithaca, NY, USA; b Department of Biomedical Sciences, College of Veterinary Medicine, Cornell University, Ithaca, NY, USA; c Division of Nephrology and Hypertension, George Washington University, Washington DC, USA; d Division of Nephrology and Hypertension, Department of Medicine, NewYork-Presbyterian-Weill Cornell Medicine, New York, NY, USA; e Genomic Resource Core Facility, Weill Cornell Medicine, New York, NY, USA; f Department of Transplantation Medicine, NewYork-Presbyterian Hospital-Weill Cornell Medicine, New York, NY, USA; g Calcium Signaling Group, Research Department, Weill Cornell Medicine Qatar, Education City, Qatar Foundation, Doha, Qatar; h Department of Systems and Computational Biomedicine, Weill Cornell Medicine, New York, NY, USA

## Abstract

Store-operated calcium entry (SOCE) plays a critical role in regulating intracellular calcium signaling and is essential for immune cell functions. SOCE blockade with the pyrazole derivative BTP2 has been explored as an anti-inflammatory strategy in preclinical models, and Zegocractin (CM4620) is under clinical investigation as a CRAC channel inhibitor with activity in multiple tissues, including immune cells. However, the cell type–specific consequences of SOCE blockade under defined activation contexts remain incompletely understood. Here, we used multiplexed single-cell RNA sequencing to investigate the effects of two prototypic SOCE blockers, BTP2 and CM4620, on polyclonally stimulated, normal human peripheral blood mononuclear cells (PBMCs) in a phytohemagglutinin (PHA)-driven T-cell activation model. The data revealed that SOCE blockade suppressed cytotoxicity-associated transcriptional programs in CD8^+^ effector T cells and natural killer (NK) cells, restoring them to levels comparable to unstimulated cells. At the same time, SOCE blockade allowed CD4^+^ regulatory T cells to retain transcriptional signatures associated with immune regulation. These results indicate that, in this experimental model, SOCE blockade dampens cytotoxic programs while maintaining tolerance signatures, suggesting a potential avenue for targeted immune modulation in transplantation and other immune-mediated conditions.

## 1. Introduction

Calcium (Ca^2+^) is an important second messenger and is critical for cellular function [1]. The activation of membrane phospholipase C, followed by the generation of inositol triphosphate (IP_3_) and its binding to receptors on the endoplasmic reticulum (ER), triggers the release of Ca^2+^ from the ER into the cytosol [2]. Depletion of Ca^2+^ from the ER induces Stromal Interaction Molecule (STIM) proteins to undergo conformational changes and cluster at ER–plasma membrane (PM) junctions, where they recruit Orai channel family members to mediate store-operated calcium entry (SOCE), resulting in a sustained increase in cytosolic Ca^2+^ [3,4]. Calcium influx through Calcium Release-Activated Calcium (CRAC) channels is a critical signaling event in immune cell activation and function of T cells and other immune subsets [5–7]. It is well known that the dephosphorylation of Nuclear Factor of Activated T cells (NFAT) by calcium-calmodulin-activated calcineurin exposes the nuclear localization sequence, facilitating NFAT transport into the nucleus [8]. In CD8 cytotoxic T cells, the NFAT translocation and binding to its promoter site is a non-redundant critical step in CD8 cells activation and cytolytic function [9,10]. In Regulatory T (Treg) cells, NFAT contributes to the induction of FOXP3 expression by binding to the Foxp3 enhancer in cooperation with Smad3 [11]. Additionally, NFAT cooperates with FOXP3 at the CTLA‑4 promoter, forming a FOXP3:NFAT complex, to promote CTLA‑4 transcription, a key mediator of Treg suppressive function [12].

Zegocractin or CM4620, a SOCE blocker that is undergoing phase 2 clinical trial as an anti-inflammatory agent, inhibits the calcium influx mediated by ORAI1, the pore-forming subunit of the calcium release-activated calcium (CRAC) channel [13,14]. Preclinical and clinical studies indicate that CM4620 acts on multiple tissues, including the pancreas, pulmonary endothelium, and immune cells, and its potential therapeutic applications remain under investigation [15]. Another small molecule, YM-58483 (also known as BTP2), has been widely tested in vitro as an inhibitor of SOCE. It suppresses CRAC channel currents primarily through its action on Orai1, with possible modulatory effects on STIM1, although all of its precise molecular targets remain incompletely defined [16,17]. Additionally, BTP2 has also been shown to affect TRPC channels, which are part of the transient receptor potential channel family involved in calcium entry [18]. Despite targeting multiple components of SOCE, BTP2 remains a potent SOCE inhibitor and has demonstrated efficacy in preclinical studies for its ability to suppress immune responses; however, BTP2 has not progressed to clinical trials for human use [19–22]. Previous work from our group, using bulk RNA sequencing, demonstrated that SOCE blockade, through the use of BTP2, spared activation-induced expression of anti-inflammatory genes while blocking pro-inflammatory gene expression [22]. However, bulk RNA-seq cannot resolve how distinct lymphocyte subsets respond to SOCE inhibition. Also, our earlier study did not address the effects of a newer, clinically advanced Orai1 inhibitor such as CM4620, leaving a critical gap in understanding the cell type-specific and mechanistic consequences of targeting this pathway in human immune cells.

Advances in high-throughput single-cell technologies have enabled the study of different cellular processes and pathways in diverse cell types [23,24]. In this study, we utilized multiplexed single-cell RNA sequencing to investigate and compare the effects of BTP2 and CM4620 on PBMCs from healthy donors in a phytohemagglutinin (PHA)–driven T-cell activation model. PHA was selected as a polyclonal stimulus because it robustly activates a broad repertoire of T cells and is widely used in clinical immune monitoring, including the FDA-approved ImmuKnow assay [25,26]. Using unfractionated PBMCs preserved physiologically relevant contact-dependent and cytokine-mediated interactions among T cells, NK cells, and monocyte-lineage cells that shape SOCE-dependent programs [27–29]. In contrast, anti-CD3/CD28 and antigen-specific activation systems, while valuable for targeted validation, are more variable and donor- or antigen-dependent, limiting their suitability as first-line discovery platforms in heterogeneous primary human samples [30,31]. Our analysis revealed that SOCE blockade using either BTP2 or CM4620 suppressed the expression of cytotoxicity-associated genes in CD8^+^ effector T cells and natural killer (NK) cells, restoring them to levels comparable to those in unstimulated PBMCs. Importantly, we find that these treatments spared the activation-induced expression of anti-inflammatory genes in CD4^+^ regulatory T cells, which retained a transcriptional signature consistent with immune regulation even after SOCE blockade. Taken together, these findings suggest that SOCE blockers can modulate inflammatory responses while maintaining regulatory pathways, motivating future functional studies to define their translational potential.

## 2. Results

### 2.1. RNA sequencing of PHA-stimulated human PBMCs treated with SOCE blockers

**Fig. 1.**
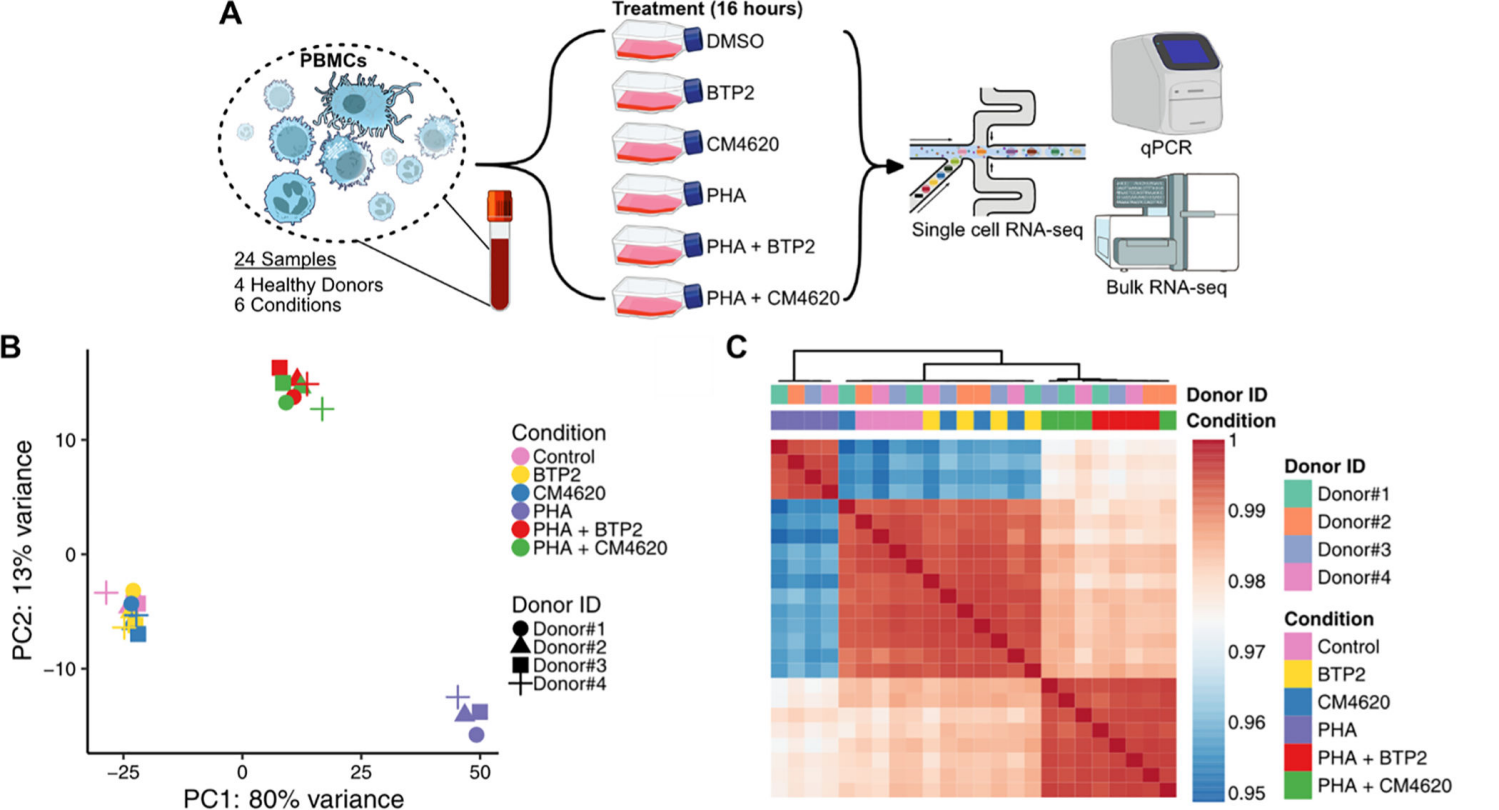
Bulk RNA sequencing of PHA-stimulated human PBMCs treated with SOCE blockers. **A)** Experimental workflow for RNA-seq of PHA-stimulated human PBMCs treated with SOCE blockers BTP2 and CM4620. **B)** Principal component analysis (PCA) plot of PBMC transcriptomes from four healthy donors colored by treatment conditions. **C)** Heatmap showing hierarchical clustering of PBMC transcriptomes from four healthy donors colored by treatment conditions.

To investigate the effect of SOCE channel blockers on diverse immune cell types including at the single cell level, we treated PHA-stimulated primary peripheral blood mononuclear cells (PBMCs) from healthy human volunteers with two SOCE channel blockers, BTP2 or CM4620 (Methods, Fig. 1A). As robust controls, we used unstimulated (no PHA) and untreated (No BTP2 or CM4620) cells, unstimulated but BTP2 treated cells, and unstimulated but CM4620-treated cells from the same donors. To investigate the transcriptional effects of SOCE channel blockers and the cell-type specificity of these effects on diverse immune cell types, we performed both bulk and single-cell RNA sequencing on human PBMCs across six conditions: (i) DMSO (Control), (ii) BTP2, (iii) CM4620, (iv) PHA, (v) PHA + BTP2, (vi) PHA + CM4620. (Fig. 1A). Supplementary Fig. S1 (Supplementary Fig. S1) shows quality control for bulk-RNA-seq samples across different donors and treatment conditions. We analyzed the bulk RNA-seq data to understand the aggregated impact of SOCE blockers on bulk transcriptomes of PHA-stimulated PBMCs. Dimensional reduction and visualization of bulk transcriptomes using Principal Component Analysis (PCA) for samples across all six treatment conditions revealed three main groups. Control PBMCs, PBMCs treated with BTP2 alone, and PBMCs treated with CM4620 alone formed a single cluster, PBMCs stimulated with PHA formed a separate cluster, and the third cluster consisted of PBMCs stimulated with PHA and treated with BTP2 and PBMCs stimulated with PHA and treated with CM4620 (Fig. 1B). We performed hierarchical clustering on batch corrected RNA-seq data and found that the samples clustered based on treatment conditions and not based on individual volunteers, confirming that the source of PBMCs had a minimal impact (Fig. 1C). Supplementary Table S1 provides the normalized base mean counts for all genes across the six experimental conditions.

We performed differential gene expression analysis (DGEA) on PBMCs stimulated with PHA and compared them to control PBMCs. DGEA revealed a significant upregulation of 3519 genes in PHA-stimulated cells (log_2_ fold-change *>* 1.0, adjusted p-value *<* 0.01, Supplementary Fig. S2A). Among these, 94 genes showing the highest fold-changes (log_2_ fold-change *>* 5.0, adjusted p-value *<* 0.01) in PHA-stimulated cells were associated with ontology terms such as cellular response to interleukin-1, response to chemokines, neutrophil chemotaxis, leukocyte proliferation and differentiation, and T cell differentiation (Supplementary Fig. S2B). Next, to understand the effect of SOCE blockers on PHA-stimulated cells, we compared significantly altered genes from PBMCs + PHA vs. PBMCs + PHA + BTP2, and significantly altered genes from PBMCs + PHA vs. PBMCs + PHA + CM4620. DGEA revealed a downregulation of 1364 and 1339 genes in PHA-activated cells when treated with BTP2 and CM4620, respectively (log_2_ fold-change *<* −1.0, adjusted p-value *<* 0.01, Supplementary Table S1). Comparison of DGEA results for PHA-stimulated cells treated with BTP2 or CM4620 revealed a high correlation between changes in transcriptional changes induced by the two SOCE entry blockers BTP2 and CM4620 (R = 0.95, p-value = 2.2e-16, Supplementary Fig. S2C). This was further supported by minimal differences observed in a direct comparison between PHA-stimulated cells treated with BTP2 and PHA-stimulated cells treated with CM4620 (Supplementary Fig. S2D, Supplementary Table S1). Our global gene expression analysis identified targeted modulation of transcriptional programs: SOCE blockade with either BTP2 or CM4620 preserved the activation-induced transcription of tolerance-associated genes (e.g., less than 50 % inhibition of activation induced expression of genes for TGFB1, IL10, and CTLA4) whereas the induced expression of proinflammatory genes such as IFNG (97 % inhibition by BTP2 and 97 % inhibition by CM4620) and cytotoxicity-associated genes such as GZMB (83 % inhibition by BTP2 and 84 % inhibition by CM4620) were markedly inhibited (Supplementary Fig. S3, Supplementary Table S1).

We utilized an orthogonal platform, the RT-qPCR assay, to validate the differential impact of BTP2 and CM4620 on PHA-induced alterations in gene expression. Our measurement of absolute mRNA copy number using the RT-qPCR assay confirmed RNA-seq identified differential impact on gene expression (Supplementary Table S2). As observed with RNA-seq, the expression level of mRNAs for the T cell receptor CD3E and T cell subtypes CD4 and CD8A were not altered by either BTP2 or CM4620 (Supplementary Fig. S4). As found using RNA seq, ITGAE mRNA was downregulated by PHA activation, and this downregulation was reversed by both BTP2 and CM4620 (Supplementary Fig. S4). In accord with RNA Seq findings, both BTP-2 and CM4620 markedly suppressed PHA-induced expression of mRNAs encoding T cell costimulatory molecules CD27 and CD28, as well as mRNAs for IL-2, CD25, proinflammatory IFNG, IFNG induced chemokines CXCL9 and CXCL10, and cytopathic proteins perforin and granzyme B. Also, as found by RNA seq, neither BTP2 nor CM4620 altered the PHA-induced increase in the expression of mRNA for prototypic immunosuppressive cytokine TGFB1. There was a modest suppression of mRNAs for IL10 and CTLA4 (Supplementary Fig. 4). Collectively, these results support at the transcript level that both BTP2 and CM4620 markedly reduce the expression of pro-inflammatory genes while relatively sparing the induced expression of tolerance-associated genes.

### 2.2. scRNA sequencing of PHA-stimulated human PBMCs treated with SOCE blockers

To investigate the cell-type specificity of the effects of SOCE channel blockers on diverse immune cell types, we performed single-cell RNA sequencing on human PBMCs across all six treatment conditions (Fig. 1A, Supplementary Fig. S5A-B). After removing cells with low mRNA counts and potential cell doublets, we detected a median of 3,484 to 4,868 transcripts across donors (Supplementary Fig. S5A). Analyzing the number of genes detected and the number of transcripts across conditions revealed a higher number of distinct genes, as well as a greater total number of transcripts, in PBMCs activated with PHA (Supplementary Fig. S5B). Interestingly, PHA-activated PBMCs treated with BTP2 or CM4620 exhibited lower transcript diversity and transcript counts compared to PHA-activated cells but still displayed higher values than control PBMCs (Supplementary Fig. S5B). Moreover, cell type proportions seemed to be fairly stable across conditions (Supplementary Fig. S5C-D). Pearson correlation analysis across samples within each donor revealed high correlations, typically ranging from 0.92 to 1.0, between technical replicates indicating strong reproducibility within each condition (Supplementary Fig. S6). Clustering patterns revealed that replicates from the same treatment condition group tightly together, further emphasizing consistency (Supplementary Fig. S6).

We analyzed single-cell transcriptomes from PBMCs (Control) condition alone to characterize the cellular heterogeneity in unstimulated (no PHA) and untreated (no BTP2 or CM4620) PBMCs (Supplementary Fig. S7A-C). We detected distinct transcriptomic clusters for various immune cell types: B cells, CD14^+^ classical monocytes, CD16 + non-classical monocytes, dendritic cells, CD8^+^ effector T cells, CD8 + naïve T cells, CD4^+^ T cells, NK cells and plasma cells (Supplementary Fig. S7A-C). As shown, we detected distinct clusters for naïve CD8 + T cells and effector CD8 + T cells, with naive CD8^+^ cells transcriptionally more similar to CD4^+^ T cells than to the effector CD8^+^ T cells (Supplementary Fig. S7A).

To analyze the effect of SOCE blockers on unstimulated PBMCs, we analyzed unstimulated PBMCs treated with BTP2 or CM4620 with control PBMCs. Unsupervised clustering on single-cell transcriptomes from these three conditions revealed distinct clusters for all immune cell types containing cells derived from all three conditions (Supplementary Fig. S7D-F). Differential gene expression analysis on BTP2 or CM4620 treated PBMCs compared to control PBMCs for each cell type showed a minimal number of significantly altered genes (Supplementary Fig. S8A-B, Supplementary Table S3–S4).

**Fig. 2.**
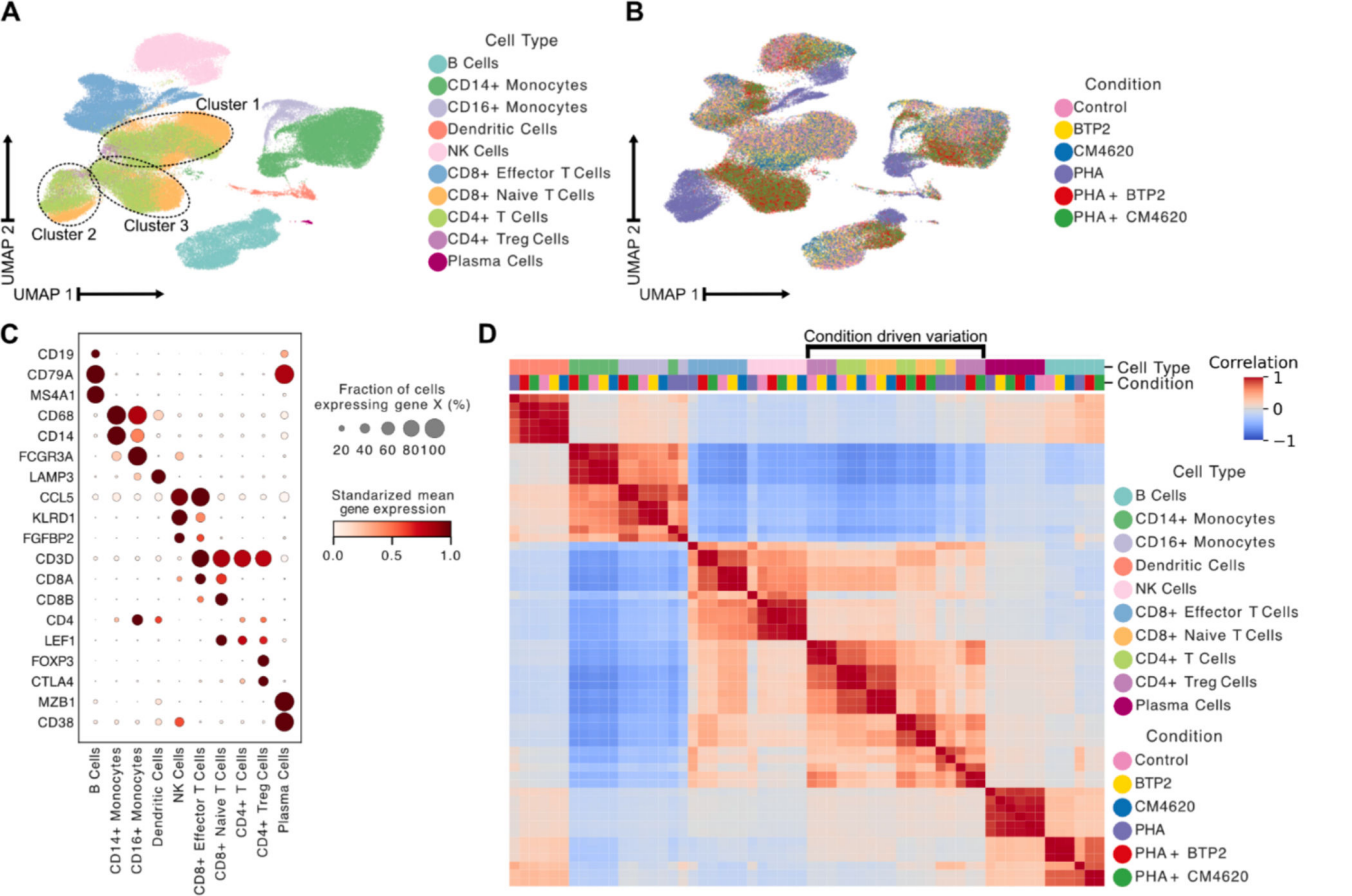
Single-cell RNA sequencing of PHA-stimulated human PBMCs treated with SOCE blockers. **A)** Uniform Manifold Approximation and Projection (UMAP) plot of single-cell transcriptomes of PBMCs from all treatment conditions clustered using gene expression and colored by cell type. **B)** Single-cell transcriptomes of PBMCs from all treatment conditions clustered using gene expression and colored by treatment conditions. **C)** Dot plot showing normalized expression of canonical cell type markers across all cell types. **D)** Heatmap showing pairwise Pearson correlation of single-cell transcriptomes of all cell types split by condition.

We analyzed single-cell transcriptomes across all six conditions together to understand the effect of BTP2 and CM4620 on PHA-stimulated cells. We clustered the transcriptomes from these six conditions to label populations of all immune cell types. We observed separation of cells from treatment conditions within clusters representing all major cell types: B cells, CD14^+^ classical monocytes, CD16 + non-classical monocytes, dendritic cells, NK cells, CD8^+^ effector T cells, CD8 + naïve T cells, CD4^+^ T cells, CD4 +Treg cells, and plasma cells (Fig. 2A-C, Supplementary Fig. S9). Interestingly, CD8^+^ Naïve T cells, CD4^+^ T cells, as well as CD4^+^ T-reg cells separated into three distinct clusters based on treatment conditions: Cluster 1 containing unstimulated and untreated cells, unstimulated and BTP2 treated cells, and unstimulated and CM4620-treated cells, Cluster 2 containing PHA-stimulated cells, and Cluster 3 containing PHA-stimulated and BTP2 or CM4620-treated cells (Figs. 2A & 2B). Cell type annotations were corroborated using the automated annotation framework CellTypist (Supplementary Fig. S10). To quantitatively assess the effect of PHA activation and the effect of BTP2 and CM4620 on PHA-stimulated cells, we performed differential gene expression analysis on individual cell types to compare PHA-stimulated PBMCs to control unstimulated PBMCs (Supplementary Fig. S11A & Supplementary Table S5), and to compare PHA-stimulated PBMCs treated with SOCE blockers to PHA-stimulated PBMCs but untreated with SOCE blockers (Supplementary Fig. S11B-C & Supplementary Table S6–S7). To assess the effect of PHA activation and the effect of BTP2 and CM4620 on PHA-stimulated cells across the entire transcriptome, we next estimated pairwise Pearson correlations for all cell types across all six conditions. Correlation analysis revealed groups of cell types for almost all cell types except CD4^+^ T cells, CD8^+^ Naïve T cells, and CD4^+^ T-reg cells, suggesting that these cell types showed the biggest changes in transcription on PHA activation and after treatment with SOCE blockers. (Fig. 2D).

### 2.3. Effects of BTP2 and CM4620 on transcriptional programs in human PBMCs

**Fig. 3.**
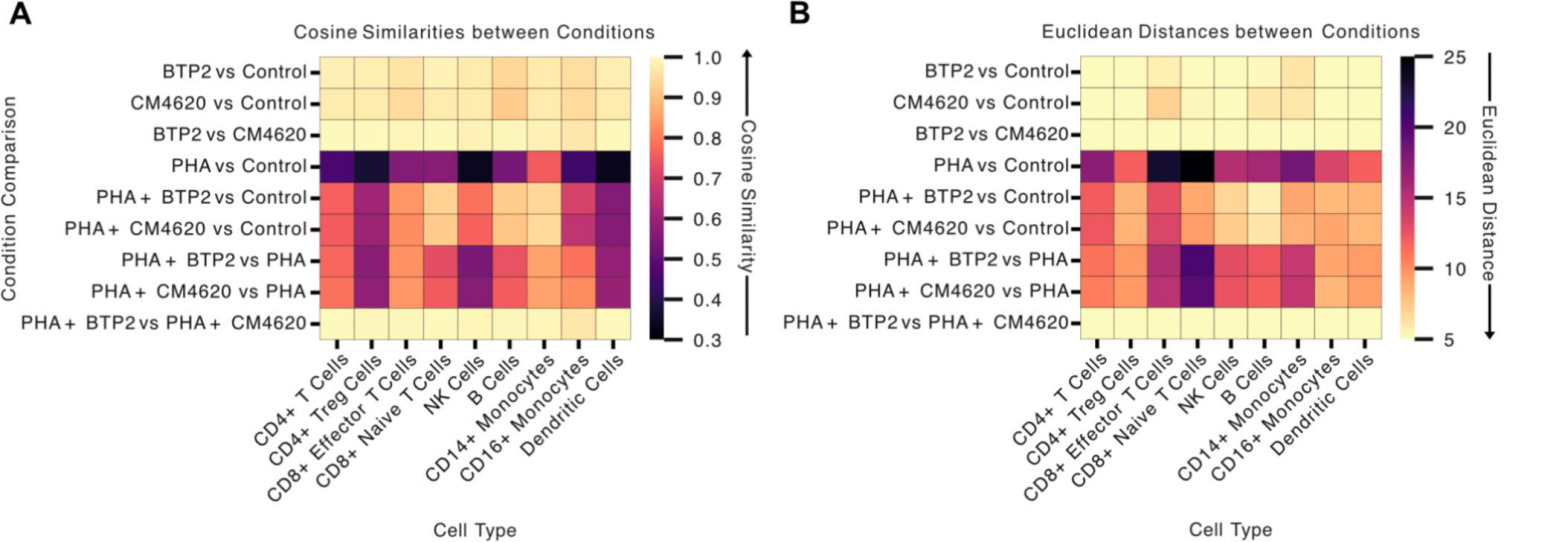
Similarity metrics and DGE analysis on cells treated with the two SOCE blockers: BTP2 and CM4620. Heatmaps show the **A)** cosine similarity and **B)** Euclidean distance between pairs of treatment conditions for each cell type.

To investigate if the two SOCE blockers have a similar effect on the transcriptomes of different immune cell types, we calculated the cosine similarity score and Euclidean distance between pairs of conditions for each cell type (Methods). We used cosine similarity as a metric for similarity in gene programs that are impacted and Euclidean distance as a measure of similarity in the magnitude of gene expression. The unstimulated controls had the highest cosine similarity scores and lowest Euclidean distances across all cell types (Fig. 3A-B). This is consistent with the observation that the unstimulated controls cluster together on the embedded space. As expected, we observed significantly lower cosine similarity and a higher Euclidean distance when comparing PHA-stimulated samples to controls or PHA-stimulated BTP2 or CM4620 treated samples to PHA-stimulated untreated samples (Fig. 3A-B).

Cosine similarity (pattern) and Euclidean distance (magnitude) provided complementary views of drug effects. In CD8^+^ effector T cells and NK cells, PHA vs. PHA + inhibitor comparisons showed relatively higher cosine similarity but larger Euclidean distances as compared to Treg cells, consistent with dampening of the same cytotoxic program (that is, similar direction but reduced scale). In contrast, Tregs exhibited lower cosine similarity but smaller Euclidean distances, indicating broader reweighting of transcriptional programs with limited overall magnitude change. This pattern is consistent with our hypothesis that cytotoxic associated transcriptional programs are strongly reduced in CD8/NK, whereas Tregs retain elevated tolerance-associated molecular programs despite transcriptome-wide remodeling.

Interestingly, we observed that the two conditions- PHA + BTP2 and PHA + CM4620- demonstrate high similarity based on both metrics, suggesting that the treatment with BTP2 or CM4620 elicit similar transcriptional changes both in terms of gene programs that were affected but also the magnitude of change in the expression of these genes. To further check if there are any differences between BTP2 and CM4620, we performed a direct DGEA between unstimulated but BTP2 treated PBMCs and unstimulated but CM4620 treated PBMCs for each cell type, as well as between the PHA-stimulated cells treated with the BTP2 or CM4620 for each cell type. This analysis revealed no significant (+/−1.0 shrunken log_2_ fold-change at 0.01 adjusted p-value) gene expression differences between the cells treated with the two drugs, BTP2 and CM4620, irrespective of their activation state. Overall, we find that BTP2 and CM4620, through their shared ability to target Orai family members-particularly Orai1- mediated highly similar transcriptional effects on human PBMCs.

### 2.4. Impact of SOCE blockers on inflammatory and anti-inflammatory transcriptional programs

Given the impact of SOCE blockade on transcription factors crucial for T cell activation and cytokine release, we next characterized, at the single-cell level, how BTP2 and CM4620 influence the inflammation-associated molecular pathways in PHA-stimulated T cells. To first characterize the phenotype of PHA-stimulated cytotoxic T cells, we compared PHA-stimulated T cells to unstimulated controls. DGEA revealed a significant upregulation of 962 genes and a significant downregulation of 828 genes in PHA-stimulated CD8^+^ Effector T cells and upregulation of 651 genes and a significant downregulation of 553 genes in PHA-stimulated NK cells (|shrunken log_2_ fold-change| *>* 1.0 and adjusted p-value *<* 0.01, Supplementary Fig. S12A & B, Supplementary Table S8–S9). Gene ontology analysis of the most significantly upregulated (shrunken log_2_ fold-change *>* 1.0 and adjusted p-value *<* 0.01) genes in CD8^+^ Effector T cells revealed an enrichment of pathways such as leukocyte mediated cytotoxicity and T cell mediated cytotoxicity (Supplementary Fig. S12C). In NK cells, gene set enrichment analysis of upregulated (shrunken log_2_ fold-change *>* 1.0 and adjusted p-value *<* 0.01) genes revealed an enrichment of ontology terms such as leukocyte mediated cytotoxicity and regulation of leukocyte mediated cytotoxicity (Supplementary Fig. S12D).

**Fig. 4.**
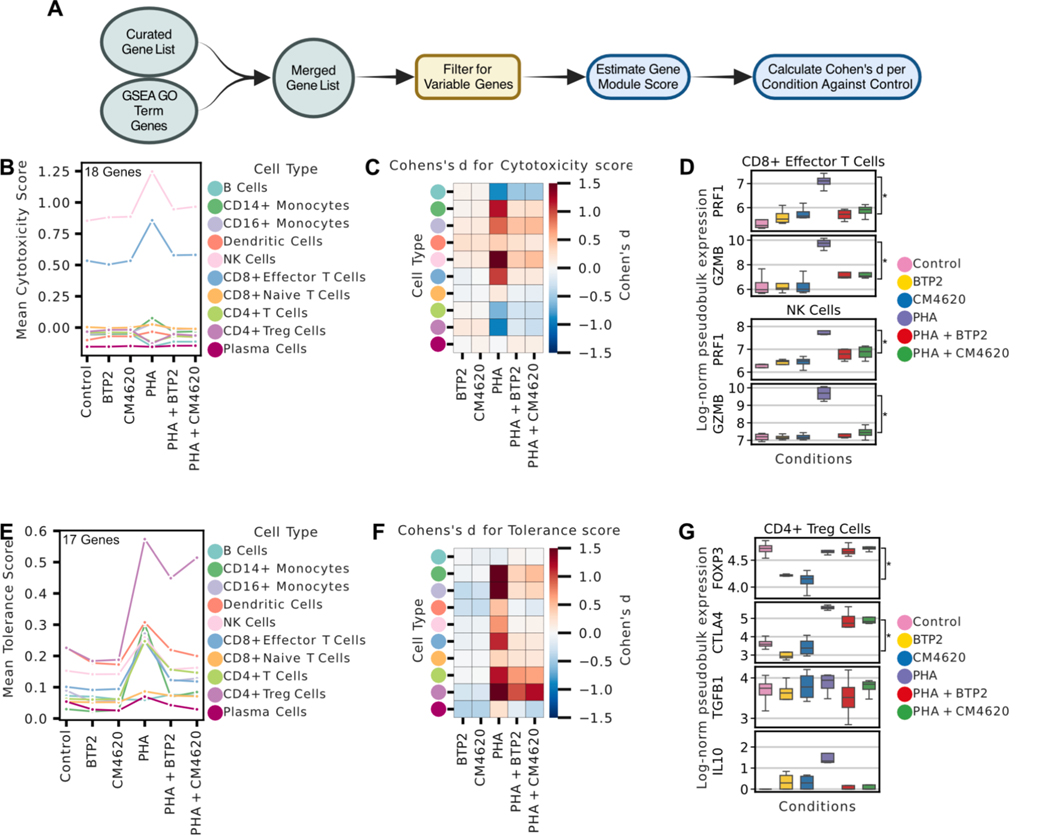
Comparison of cytotoxic and tolerance gene expression in PHA-stimulated PBMCs treated with SOCE blockers. **A)** Flowchart overview of analysis for estimating gene module scores and Cohen’s *d* metric between conditions. **B)** Line plot showing the mean cytotoxicity scores, generated using 18 cytotoxicity genes, for each cell type split across treatment conditions. **C)** Heatmap of Cohen’s *d* metric for cytotoxicity for each cell type comparing each condition against the PBMCs (Control) condition. **D)** Box plots showing log transformed normalized pseudobulk expression of GZMB and PRF1 in CD8^+^ Effector T cells and NK cells split by the six condition groups. Asterisks denote significantly altered groups between conditions (adjusted p *<* 0.01). **E)** Line plot showing the mean tolerance scores, generated using 17 tolerance genes, for each cell type split across treatment conditions. **F)** Heatmap of Cohen’s *d* metric for tolerance for each cell type comparing each condition against the PBMCs (Control) condition. **G)** Box plots showing log transformed normalized pseudobulk expression of FOXP3, CTLA4, TGFB1, and IL10 in CD4^+^ Treg cells split by the six condition groups. Asterisks denote significantly altered groups between conditions (adjusted p *<* 0.01).

We then generated gene programs using a curated list of 18 genes found in T cell cytotoxicity associated gene ontology terms and used it to estimate a cytotoxicity transcriptional module score for all cells across the six conditions (Methods, Fig. 4A, Supplementary Fig. S13A & B). Comparison of mean cytotoxicity scores for NK cells and all T cell types across four treatment conditions showed that the score was increased in NK cells and in CD8^+^ effector T cells following stimulation of PBMCs with PHA (Fig. 4B). Both BTP2 and CM4520 decreased the cytotoxicity score in NK cells and CD8 + effector T cells, and the scores were similar to control cells (Fig. 4B). This new finding, identified by scRNA sequencing, confirms and extends our previous finding observed by bulk RNA-seq of PBMCs [22]. We calculated Cohen’s *d* metric for all conditions when compared to unstimulated control and found that for NK cells and effector CD8^+^ T cells, Cohen’s *d* was maximum for PHA-stimulated cells and the score subsequently decreased for PHA-stimulated cells treated with BTP2 or CM4620 (Fig. 4C). Visualizing the pseudobulk expression GZMB and PRF1 specifically, their expression is significantly elevated in PHA stimulated PBMCs followed by a significant decrease after drug treatment in both CD8^+^ effector T cells and NK cells (Fig. 4D). This is consistent with both bulk RNA-seq and RT-qPCR results (Supplementary Fig. S3F & 4F).

Next, we generated a tolerance/anti-inflammatory gene module using a curated list of 17 genes in ontology terms associated with tolerance and anti-inflammatory pathways (Fig. 4A, Supplementary Fig. S13C & 13D). We then compared tolerance gene module scores across all six conditions for all immune cell subtypes. Stimulation of PBMCs with PHA resulted in an increase of anti-inflammatory/tolerance gene expression and an increase in tolerance module score in CD14^+^ classical monocytes, CD16 + non-classical monocytes, dendritic cells, NK cells, CD8^+^ effector T cells, CD4^+^ T cells, and CD4 + Treg cells but not in CD8^+^ Naive T cells, B cells, and plasma cells (Fig. 4E). Importantly, CD4^+^ T-reg cells were the only PHA-stimulated cell type that retained elevated tolerance-associated transcriptional module scores after treatment with either BTP2 or CM4620. All other immune cells demonstrated a decrease in tolerance module score after treatment with BTP2 or CM4620 (Fig. 4E). We then calculated Cohen’s *d* metric for each condition compared to the unstimulated untreated condition for CD4^+^ T-reg cells and found that the Cohen’s *d* metric was large not only for the PHA stimulated cells, but also for the stimulated cells treated with BTP2 or CM4620 (Fig. 4F). Consistent with this, the expression of CTLA4 is increased in stimulated PBMCs and maintained at a level higher than the controls even after BTP2 or CM4620 treatment in CD4^+^ Treg cells (Fig. 4G). It is important to note that bulk RNA-seq and qPCR analyses showed modest reductions in FOXP3, CTLA4, and IL10 expression after SOCE blockade (Supplementary Fig. S3 & S4). We interpret this difference as reflecting the averaging of mixed PBMC populations in bulk assays, where changes in non-Treg lineages can obscure Treg-specific expression. Furthermore, we note that TGFβ1 and IL10 transcripts are captured at very low levels in our single-cell RNA-seq dataset, which likely reflects technical limitations of scRNA-seq rather than biological suppression. This explains the apparent discrepancy with bulk RNA-seq and qPCR, which detect modest reductions in these transcripts after SOCE blockade. However, at a broader level, the bulk RNA-seq data show significant consistency with the single-cell results for genes involved in T cell expansion, recruitment, activation, effector function, and tolerance, providing valuable orthogonal validation of our findings (Supplementary Fig. S14A-D). Together, these data indicate that despite technical limitations in capturing TGFβ1 and IL10 transcripts, bulk and single-cell analyses show strong agreement across key T cell functional programs and that tolerance-associated module scores remain relatively high in Tregs compared to other lineages.

Furthermore, we performed DGEA for PHA-stimulated CD4^+^ T-reg cells treated with BTP2 or CM4620 compared to unstimulated control (Supplementary Table S10–S11). The DGEA analysis revealed upregulated (shrunken log_2_ fold-change *>* 1.0 and adjusted p-value *<* 0.01) genes associated with ontology terms such as regulation of T cell activation for both drugs (Supplementary Fig. S15). The GO term for regulation of T cell activation in the PHA-stimulated CM4620-treated PBMCs showed an enrichment in 20 genes: ACTB, IRF4, PNP, CD40LG, HSPH1, HSPD1, LGALS1, SIRPG, DUSP10, BATF, SELENOK, PTPN6, CTLA4, IL4I1, YES1, TNFRSF9, LRRC32, TFRC, XBP1, and CORO1A, many of which are known to be associated with T-reg cell tolerance. Similarly, for the same GO term, PHA-stimulated BTP2-treated cells showed an enrichment in genes such as ACTB, IRF4, PNP, CD40LG, HSPH1, HSPD1, IL23A, RAC2, LGALS1, SIRPG, BATF, SLC7A1, SELENOK, PTPN6, CTLA4, IL4I1, YES1, TNFRSF9, LRRC32, XBP1, and CORO1A, many of which are associated with T-reg cell tolerance. Overall, we report that transcriptional signatures associated with Treg-mediated tolerance were retained in PHA-stimulated PBMCs after SOCE blockade, whereas cytotoxicity-associated gene programs were reduced.

## 3. Discussion

This study extends prior bulk RNA sequencing work on BTP2 in human PBMCs by providing single-cell resolution of SOCE inhibitor effects and by incorporating CM4620, a clinically advanced Orai1 inhibitor, into the analysis. By mapping BTP-2- and CM4620-induced transcriptional programs across NK cells, CD8 + effector T cells and regulatory T cells, our data reveal subset-specific sensitivity to SOCE blockade and demonstrate preferential attenuation of cytotoxic and effector gene modules with preservation of regulatory transcriptional programs, thereby offering a more granular mechanistic framework for how SOCE inhibitors may achieve selective immunomodulation in humans.

SOCE blockade has been explored extensively across mechanistic, preclinical and early translational studies with consistent evidence that CRAC channel inhibition suppresses inflammatory effector responses. Functional genetics and pharmacological studies established that STIM-ORAI-mediated SOCE is the dominant calcium influx pathway required for T-cell activation, cytokine production, and cytotoxicity [32,33]. Early studies with BTP2 showed that this potent inhibition of CRAC currents, leading to reduced interleukin and interferon gamma production and impaired T cell expansion [34].

Our findings are consistent with and extend disease-focused studies demonstrating the therapeutic potential of SOCE inhibition. In inflammatory bowel disease models, SOCE blockade reduces production of IL-2, IL-4, IL-6, IL-17A, TNF, and IFN-γ across multiple immune lineages while ameliorating colitis severity without compromising epithelial integrity [35]. In experimental pancreatitis, CM4620 reduces pathological calcium influx, acinar cell injury, NFAT and NF-κB activation, and inflammatory cytokine production, while dampening neutrophil and PBMC inflammatory responses [13]. Together, these studies support a model in which SOCE inhibition broadly suppresses pathological inflammation without inducing global immune paralysis.

Our earlier bulk RNA-seq work showed that BTP2 suppresses transcriptional programs associated with T cell activation, proliferation, cytotoxicity, and inflammatory chemokine production, while relatively preserving tolerance-associated genes. The present scRNA-seq analysis refines this view by demonstrating that SOCE inhibition is not uniform across immune compartments. NK cells and CD8^+^ effector T cells exhibit profound transcriptional suppression, whereas regulatory T cell programs are comparatively spared. This observation aligns with emerging evidence that thymic and induced Tregs exhibit weaker SOCE and NFAT activity than conventional T cells, and that limiting sustained NFAT signaling stabilizes FOXP3 expression and the Treg phenotype [36].

Mechanistically, these findings are consistent with the dual role of NFAT in effector versus regulatory T cells. In CD8^+^effector T cells, NFAT cooperates with AP-1 to drive glycolytic metabolism, CXCR3-mediated trafficking, and cytolytic programs including GZMB and PRF1 [9,37,38]. In contrast, in Tregs, NFAT forms cooperative complexes with FOXP3 to maintain tolerance-associated genes such as CTLA4, requiring only modest NFAT activity [37,39,40]. SOCE blockade therefore converges on NFAT-dependent transcription while differentially impacting effector and regulatory programs, providing a plausible mechanistic basis for selective immunomodulation.

The preferential attenuation of cytotoxic transcriptional programs has potential implications for transplantation and inflammatory organ injury. GZMB and PRF1 are highly expressed in rejecting human allografts, whereas preservation of FOXP3- and CTLA4-associated programs has been linked to reversal of T cell–mediated rejection and operational tolerance [41–43]. Our observation that CM4620 suppresses proinflammatory transcriptional circuits while relatively sparing regulatory modules provides mechanistic support for its ongoing clinical evaluation in acute pancreatitis (CARPO trial) and pancreatitis-associated acute kidney injury (KOURAGE trial [44]).

Several limitations should be acknowledged. First, PHA is a strong, non-physiological polyclonal activator that exaggerates TCR-proximal calcium influx and NFAT signaling, particularly in CD8^+^ effector T cells. Accordingly, these data should not be over-extrapolated to antigen-specific immune responses. Rather, the PHA-stimulated PBMC system should be viewed as a standardized, high-signal discovery platform for identifying core SOCE-dependent transcriptional programs within a complex human immune environment. Second, BTP2 and CM4620 produced highly correlated transcriptional effects in this system, consistent with shared SOCE inhibition but limiting resolution of compound-specific differences. The study was designed to minimize technical confounding, and we therefore interpret the results as defining a common SOCE-blockade transcriptional signature rather than differential drug behavior.

Finally, this study was explicitly optimized for high-dimensional single-cell transcriptomic discovery rather than parallel functional assays. Follow-up studies will be required to directly assess CD8^+^ T cell and NK cell cytotoxicity, Treg suppressive capacity, calcium flux dynamics, and protein-level validation of key gene modules. We view the present work as a foundational single-cell atlas that defines SOCE-dependent immune programs and provides a rational framework for targeted functional validation and therapeutic translation.

## 4. Materials and Methods

### 4.1. Healthy volunteers

Peripheral venous whole blood was obtained from healthy volunteers after obtaining informed written consent. The research project #1208012870 was approved by the Weill Cornell Medicine Institutional Review Board. The authors confirm that all research was performed in accordance with relevant guidelines/regulations, and the research involving human research was performed in accordance with the Declaration of Helsinki.

### 4.2. PBMC isolation and stimulation

Human peripheral blood mononuclear cells (PBMCs) were isolated from 4 healthy volunteers (mean age 27.75 ± 2.36 years, 3 males, 1 female, no comorbidities) using Ficoll-Paque^™^ density gradient centrifugation. PBMCs (1 × 10^6^ cells/mL) were incubated under various conditions, including stimulation with PHA (ThermoFisher, cat# R30852801) and treatment with BTP2 (Millipore Sigma, cat# 203890–5 mg) or CM4620 (MCE, cat# HY-101942) for 16 h. BTP2 and CM4620 were each used at 1 μM, a concentration widely employed to achieve near-maximal SOCE inhibition in lymphocytes without inducing overt cytotoxicity. Control samples were treated with DMSO (Gibco, cat# 14190–136). All treatment conditions for a given donor were processed in parallel from cell isolation through RNA extraction to minimize batch effects. Details of the procedure are provided in the Supplemental Methods.

### 4.3. RNA isolation and quantitative PCR

Total RNA was extracted from PBMCs using the RNeasy Mini Kit (Qiagen), followed by quantification and quality assessment. Reverse transcription and quantitative PCR (RT-qPCR) were performed to measure absolute mRNA copy numbers. Additional details are included in the Supplemental Methods.

### 4.4. Bulk and scRNA sequencing

For bulk RNA sequencing, RNA samples were treated with DNase before library preparation using the TruSeq RNA Library Prep Kit (Illumina) and sequenced on a NovaSeq 6000 system. Single-cell RNA sequencing (scRNA-seq) was performed using the 10X Genomics 3′ Gene Expression platform, with library preparation and sequencing conducted at the Weill Cornell Medicine Genomics Core. For scRNA-seq, samples were multiplexed using 10X CellPlex and pooled prior to sequencing, with randomized lane assignments to reduce technical confounding. Additional processing details are provided in the Supplemental Methods.

### 4.5. Bulk and scRNA-seq Data processing and analysis

Bulk RNA-seq reads were aligned to the human genome (GRCh38) using STAR, and gene expression counts were processed with DESeq2 for normalization and differential expression analysis [45–49]. scRNA-seq data were preprocessed using Scanpy, with quality control, normalization, clustering, and annotation based on canonical immune cell markers [50]. Immune cell annotations were independently corroborated using CellTypist with both low- and high-resolution immune reference models to assign broad and fine-grained immune cell identities [51]. Details of filtering criteria, clustering parameters, and computational tools are included in the Supplemental Methods.

### 4.6. Gene expression Similarity, effect Size Estimation, and enrichment analysis

To assess similarity between treatment conditions, Pearson correlation, Euclidean distances, and cosine similarity metrics were computed. Cosine similarity captures directional concordance of gene-expression vectors (pattern of relative gene weights), whereas Euclidean distance captures scale differences (overall magnitude); we report both to distinguish directional reweighting from amplitude changes. To quantify the effect of treatments on gene expression profiles, Cohen’s *d* was calculated for key gene modules, including cytotoxicity and tolerance scores. Differential expression was assessed on pseudobulked counts using DESeq2 (via PyDESeq2[52]), applying gene filtering, normalization, dispersion estimation, Wald tests with Benjamini–Hochberg FDR correction, and log2 fold-change shrinkage to obtain robust results. Gene set enrichment analysis (GSEA) was performed using GSEApy to identify pathways associated with cytotoxicity and tolerance [53–56]. To compare gene expression across bulk RNA-seq and single-cell RNA-seq data (donor-level pseudobulk), we calculated Cohen’s *d* and Cliff’s delta for between-condition comparisons and evaluated cross-modality correlations using Pearson and Spearman statistics. Further details are provided in the Supplemental Methods.

## Supplementary Material

1

2

3

4

5

6

7

8

9

10

11

12

## Significance Statement:

Store-operated calcium entry (SOCE) is essential for immune activation, but broad immunosuppression can cause significant side effects. Using single-cell transcriptomics in a phytohemagglutinin (PHA)–driven T-cell activation model, we show that SOCE blockade with BTP2 or CM4620 suppresses pro-inflammatory and cytotoxic programs in CD8^+^ effector T cells and NK cells while preserving tolerance-associated pathways in CD4^+^ regulatory T cells. These findings suggest that SOCE blockade may provide a more targeted form of immune modulation, warranting future head-to-head comparisons with conventional immunosuppressants. Our results highlight the potential of SOCE blockers to reduce immune-mediated damage while maintaining tolerance, motivating further functional and translational studies in transplantation and other immune-mediated conditions.

## Appendix A. Supplementary data

Supplementary data to this article can be found online at https://doi.org/10.1016/j.humimm.2026.111687.
