# Supplementary material for: Single-cell transcriptomics reveals targeted modulation of inflammatory repertoire by SOCE blockers
Source: Hum Immunol. Author manuscript; Available in PMC 2026 Aug 17. (PMC13478930; doi:10.1016/j.humimm.2026.111687)
Supplement: Supplementary file 1 [file NIHMS2189444-supplement-1.pdf]

SUPPLEMENTAL METHODS

PBMC Isolation and Stimulation

Human peripheral blood mononuclear cells (PBMCs) were isolated from 4 healthy volunteers (mean age 27.75±2.36 years, 3 males, 1 female, no comorbidities) using Ficoll-Paque (Cytiva, cat# 17544202), washed twice with Dulbecco's Phosphate-Buffered Saline (DPBS) (GIBCO, cat# 14190-136), resuspended in RPMI 11640 (Gibco, cat# 22400-089) supplemented with 5% Heat Inactivated Fetal Bovine Serum (HI FBS) (Gibco, cat# 10082-147), 100 U/ml Penicillin-Streptomycin (GenClone, cat# 25-512) and 2 mM L-Glutamine (Gibco, cat# 25030-081). The PBMCs were counted and adjusted to a concentration of 1 x 10^6 per 1 ml of complete medium and incubated for 16hrs at 37C in 95% air and 5% CO_2 for each treatment condition: PBMCs + DMSO, PBMCs + BTP2, PBMCs + CM4620, PBMCs + PHA, PBMCs + PHA + BTP2, PBMCs + PHA + CM4620. DMSO (Gibco, cat# 14190-136) was used as the solvent for BTP2 (Millipore Sigma, cat# 203890-5mg) and CM4620 (MCE, cat# HY-101942), therefore it was also added to the control PBMCs and PBMCs stimulated with 2ug/ml purified phytohemagglutinin (PHA) (ThermoFisher, cat# R30852801). This product is a freeze-dried, highly refined fraction derived from Phaseolus species seed extracts. During purification, the specific mitogenic activity and the ratio of mitogenic to hemagglutinating activity was enhanced approximately 100-fold, resulting in a preparation optimized for use as a mitogen.

Prior to mRNA gene expression, bulk RNA and scRNA assays, the PBMCs were washed once with RPMI 1640 (Gibco, cat# 22400-089) after 16 hrs. incubation.

RNA Extraction

PBMCs were washed with 1000 µl DPBS and centrifuged at 13,400 g for 2 minutes at room temperature. The supernatant was discarded, and 50ul RNAlater (Invitrogen, cat# AM7020) and Buffer RLT (Qiagen, cat# 79216) with 350 µl β-mercaptoethanol (Sigma-Aldrich, cat# M6250) were added to each cell pellet. Total RNA was isolated from each cell lysate using the RNeasy mini kit, according to the manufacturer's protocol (Qiagen, cat# 74106). The quantity (absorbance at 260 nm) and purity (ratio of 260 nm to 280 nm) of the RNA were measured using the NanoDrop® One UV-Vis spectrophotometer (Thermo Scientific).

Reverse Transcription and Quantitative PCR on PBMC RNA

The extracted RNA was reverse transcribed to cDNA using TaqMan reverse transcription kit (Applied Biosystems, cat# N808-0234), and the cDNA were pre-amplified using Platinum Multiplex PCR Master Mix (Applied Biosystems, cat # 4464269). The absolute copy number of mRNAs was quantified using the preamplification enhanced Realtime-qPCR assays[22]. The sequence and location of the oligonucleotide primers and gene specific TaqMan probes designed at our Gene Expression Monitoring Core, NY to quantify the transcripts have been published[22].

Bulk RNA library preparation for RNA-seq

RNA samples were treated with DNase using RNase-Free DNase kit (Qiagen, cat# 79254) according to the manufacturer's protocol to remove DNA prior to RNA sample library preparation and sequencing. RNA library preparation and RNA sequencing were performed by the Genomics

Core Laboratory at Weill Cornell Medicine. RNA libraries were prepared using the TruSeq RNA Library Prep Kit (Illumina) and sequenced using NovaSeq 6000 System (Illumina) as 100 x 2 paired-end reads.

Sample preparation for scRNA-seq

Two replicate batches of cells for six experiment conditions were individually processed for four donors yielding a total of 48 samples. The total and live cell counts from the PBMCs were determined using the TC20 cell counter (Bio-Rad). Approximately two million cells per sample were labelled with the cell multiplexing oligos provided as a part of the 3’ CellPlex Kit Set A (10X Genomics), according to the manufacturer’s protocol. Labelled cell suspensions from individual patient replicates were counted and pooled across the six experimental conditions to produce two final pooled suspensions per patient. These pooled cell suspensions were used to generate the 3’ cell multiplexing oligo and gene expression libraries using Chromium Single Cell 3' Reagent Kits User Guide (v3.1 Chemistry Dual Index) with Feature Barcoding technology for Cell Multiplexing CG000388 Rev C (10X Genomics) and sequenced using NovaSeq 6000 (Illumina). The library preparation and sequencing were performed by Weill Cornell Medicine Genomics Core.

Bulk RNA-seq Data Processing

Raw sequencing reads were aligned to the human genome (GRCh38) using STAR (v-2.7.10b). Gene expression counts were processed with DESeq2 (v-1.44.0), where genes with fewer than ten copies across all samples were removed. Variance-stabilizing transformation (VST) was used for normalization, and batch correction was applied using limma (v-3.60.0). Principal Component Analysis (PCA) and Differential Gene Expression Analysis (DGEA) were performed using DESeq2’s Wald test, with effect size shrinkage applied using apeglm (v-1.26.1).

scRNA-seq Preprocessing and Analysis

Raw reads from both the gene-expression and cell-multiplexing oligo libraries were processed with CellRanger’s multi command, using a configuration file that included the oligo barcode table needed to demultiplex the pooled samples. Raw gene expression matrices were processed using Scanpy (v.1.10.1). Cells with fewer than 500 genes, fewer than 1,000 UMI counts, or mitochondrial content exceeding 20% were removed. Doublets were detected using Scrublet (v.0.2.3) and excluded. Data were normalized to 10,000 counts per cell and log-transformed. Highly variable genes were identified using min_mean = 0.1 and min_dispersion = 0.2. PCA was performed using the top 20 principal components, followed by Leiden clustering (resolution = 0.3) and UMAP embedding. Clusters were annotated based on canonical immune cell markers.

For T cell subpopulation analysis, CD4+ and CD8+ T cells were subsetted and reanalyzed using 12 PCs and Leiden clustering (resolution = 2.0). CD8+ T cells were further classified as effector or naïve based on cytotoxic marker expression. CD4+ regulatory T cells (T-regs) were identified using FOXP3 expression with Leiden clustering (resolution = 2.5).

Cell Type Correlations and Similarity Metrics

PBMC cell types were split by condition into 60 groups, and mean gene expression was calculated for each. Pearson correlation coefficients were computed using Pandas (v.2.2.1), and hierarchical clustering was performed using SciPy (v.1.11.4)[59] with average linkage and correlation distance metrics.

To assess similarity between conditions, centroids for each cell type were computed using mean gene expression. Euclidean distances and cosine similarity were calculated using SciPy (v-1.11.4)[59] and scikit-learn (v.1.4.2)[60], respectively. Comparisons included control vs. drug-treated conditions and between different drug treatments.

Gene Module Scores and Cohen's d Calculation

Cytotoxicity gene module scores were derived by identifying highly variable genes in CD8+ effector T cells and NK cells, supplemented with GSEA GO term "GOBP Positive Regulation of T Cell Mediated Cytotoxicity" and known markers (NKG7, IFNG, PRF1). Scanpy's score_genes function was used to compute module scores, and Cohen's d was calculated for effect size estimation.

Similarly, tolerance scores were derived from CD4+ T-reg cell genes identified using GO terms for T cell tolerance induction. IL10 was included despite low expression. Tolerance scores and Cohen's d were calculated using the same methodology.

Differential Gene Expression and Gene Set Enrichment Analysis (GSEA)

We performed differential gene expression on pseudobulked counts by summing raw UMIs per gene to generate pseudobulk samples, retaining genes with ≥10 counts in at least two pseudobulks. DESeq2 (via PyDESeq2[54]) was used with the design ~Condition, median-of-ratios normalization, dispersion estimation, and GLM fitting with Cook's distance re-fitting. Wald statistics were used to compute p-values, which were adjusted for multiple testing using the Benjamini–Hochberg FDR method. Log2 fold changes were further stabilized by shrinkage, and only datasets with two conditions and ≥2 replicates per condition were analyzed. Upregulated genes were defined as shrunken log2 fold-change > 1 and FDR adjusted p-value < 0.01, and downregulated genes as shrunken log2 fold-change < -1 and FDR adjusted p-value < 0.01. GSEA was performed using GSEApy (v.1.0.3) with the "c5.all.v2023.2.Hs.symbols.gmt" gene set. DGE and GSEA were repeated for control vs. drug-treated conditions across all cell types, with the top GO terms visualized.

Cross-Modality Effect Size and Correlation Analysis

To compare gene expression across our bulk RNA-seq and scRNA-seq data we created donor-level pseudobulk samples from the single cell data by summing the raw counts per gene within each donor. After normalization and log transformation for both bulk and pseudobulk data, we calculated Cohen's *d* and Cliff's delta for between-condition comparisons and evaluated cross-modality correlations using Pearson and Spearman statistics.

SUPPLEMENTAL FIGURES

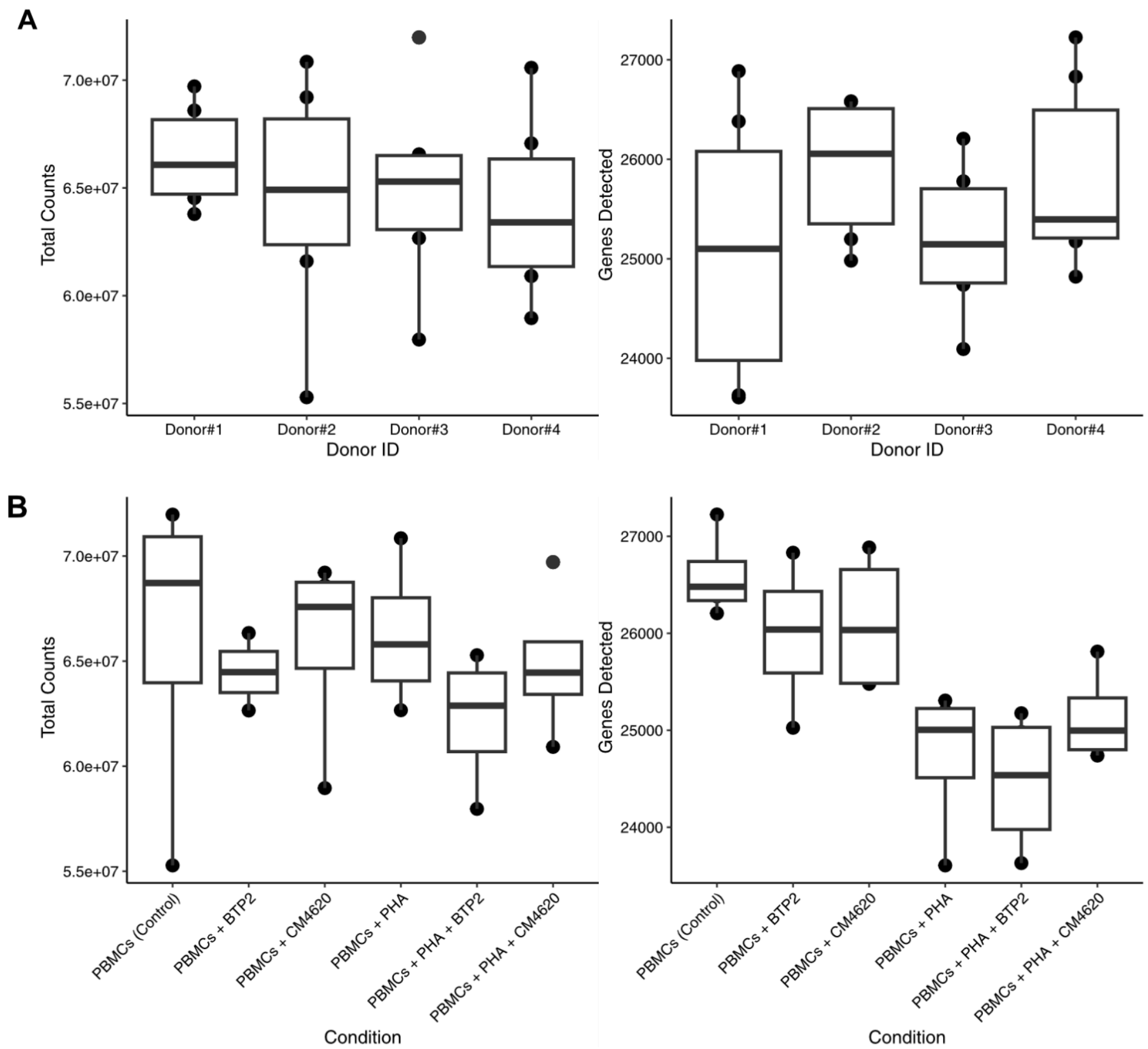

Supplementary Figure 1: **Quality control for bulk RNA-seq samples across different donors and treatment conditions. A)** Box plot showing total counts and genes detected in individual samples across donors (#1 to #4). **B)** Box plot showing total counts and genes detected in individual samples across different treatment conditions: PBMCs (Control), PBMCs treated with BTP2 or CM4620, PBMCs stimulated with PHA, and PHA-stimulated PBMCs treated with BTP2 or CM4620. Each dot represents an individual sample.

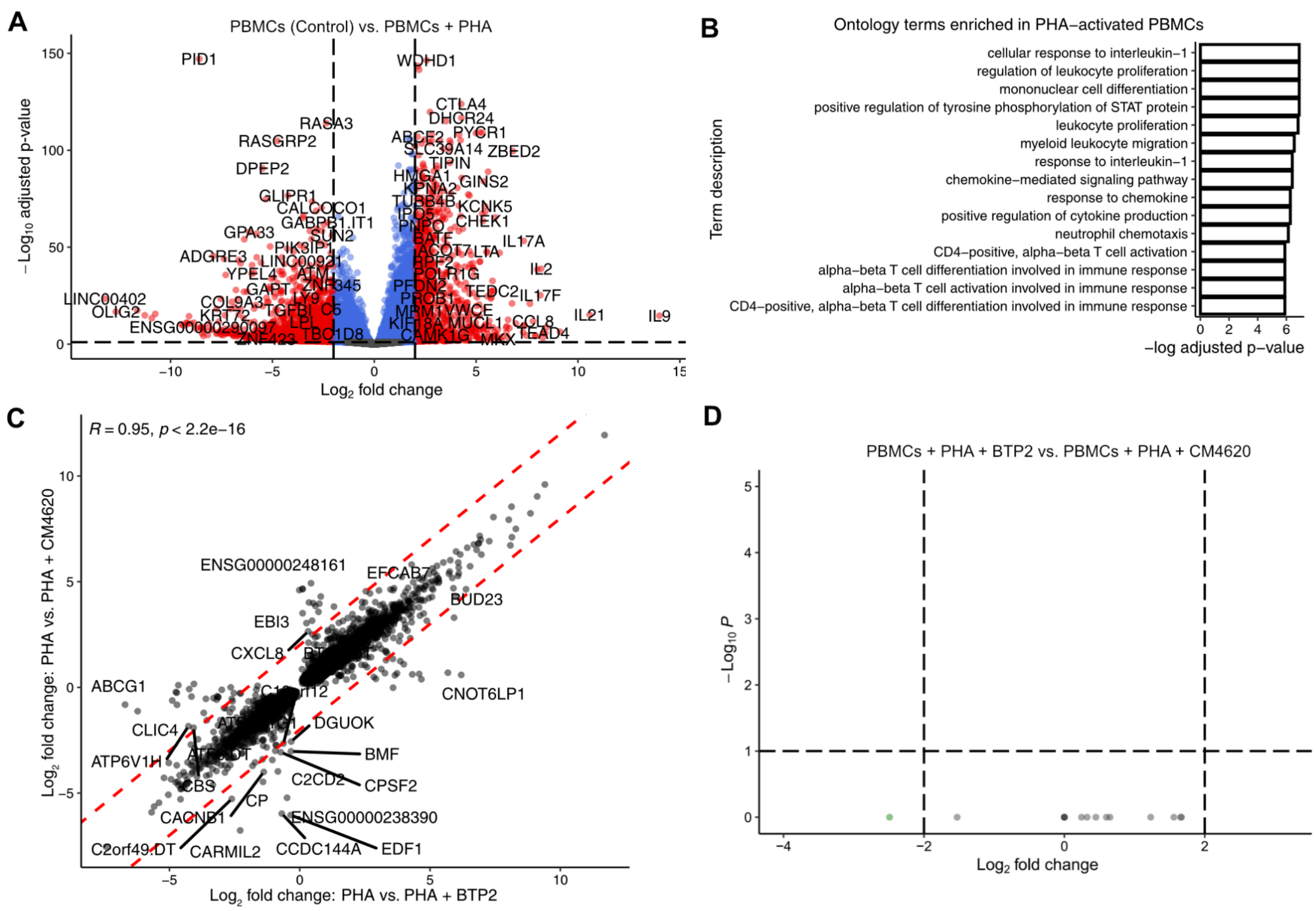

Supplementary Figure 2: **Differential gene expression analysis of bulk RNA-seq data under various conditions**. **A)** Volcano plot showing the $\log_2$ fold-change and $-\log_{10}$ adjusted p-value for genes when compared between PBMCs (Control) and PBMCs stimulated with PHA. The dotted lines define the $\log_2$ fold-change threshold of +/- 2.0 and adjusted p-value threshold of 0.01. **B)** Bar plot showing top 15 ontology terms enriched for genes upregulated in PBMCs + PHA as compared to PBMCs (Control) with $\log_2$ fold-change > 5.0 and adjusted p-value < 0.01. **C)** Scatter plot showing the correlation between log fold-change for genes significantly altered in PBMCs + PHA + BTP2 or altered in PBMCs + PHA + CM4620, as compared to PBMCs + PHA. Only significantly altered genes with adjusted p-value < 0.01 in at least one of the two comparisons were included in the plot. **D)** Volcano plot showing the $\log_2$ fold-change and $-\log_{10}$ adjusted p-value for genes when compared between PBMCs + PHA + BTP2 and PBMCs + PHA + CM4620.

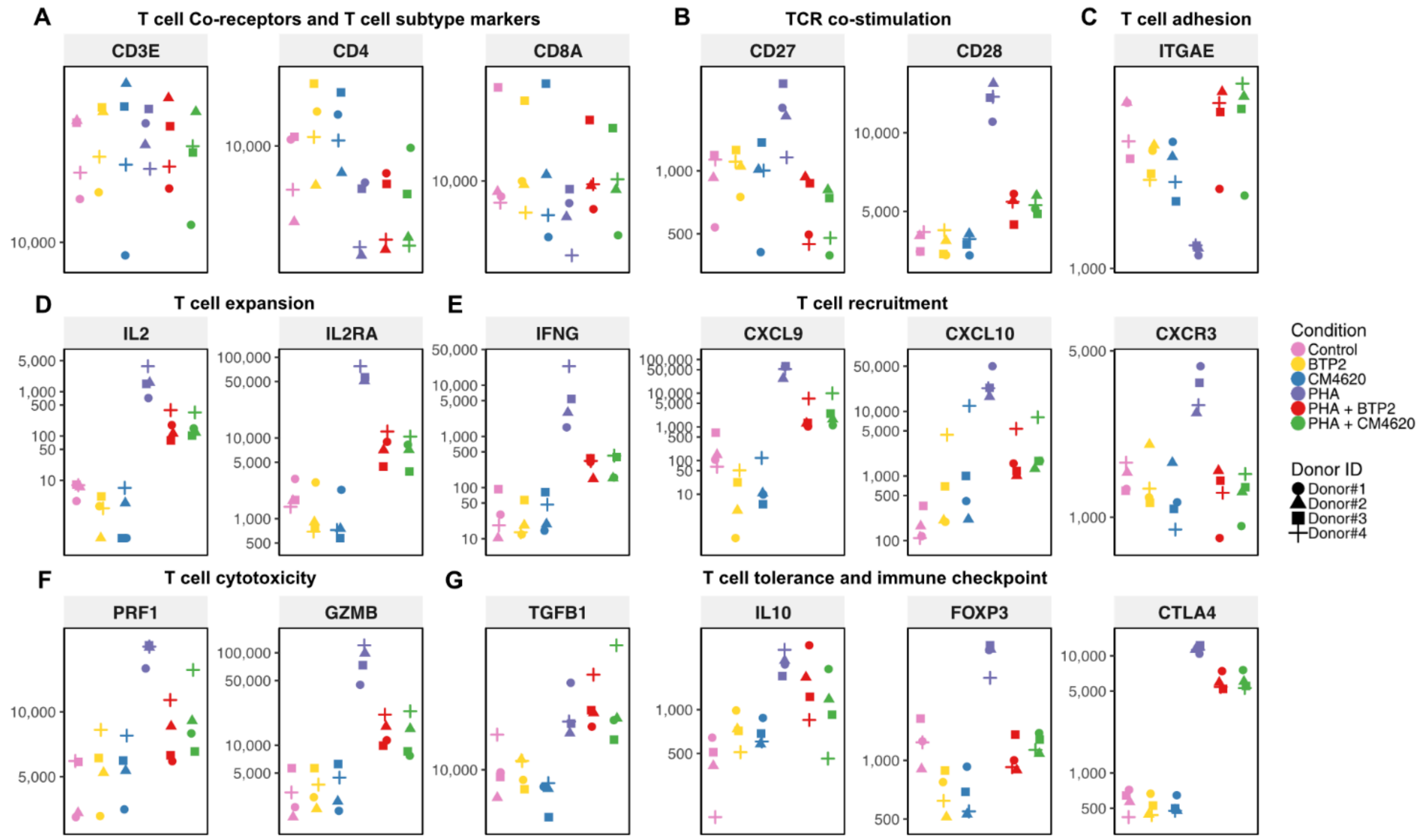

Supplementary Figure 3: Differential effects of BTP2 and CM4620 on PHA-induced alterations in the expression of mRNAs encoding proteins involved in T cells expansion, recruitment, activation, effector function, and tolerance. Jitter plots showing the log-normalized bulk RNA-seq counts across the six treatment conditions. **A)** Pan-T cell surface antigen receptor complex CD3E, CD4 expressed on T helper cells and responsible for recognition of antigenic peptide in the context of HLA class II antigens and CD8 expressed on cytotoxic T cells and responsible for recognition of antigenic peptide in the context of HLA class I antigens; **B)** T cell co-stimulation receptors CD27 and CD28; **C)** T cell adhesion marker: surface integrin ITGAE (CD103); **D)** T cell growth factor IL2 and its receptor IL2RA responsible for T cell clonal expansion; **E)** IFNG and IFNG induced T cell recruitment/homing proteins: Chemoattractant molecules and receptors: CXCL9, CXCL10, and CXCR3; **F)** T cell cytotoxic proteins PRF1 and GZMB **G)** Immunosuppressive cytokines TGFB1 and IL10, Treg cell marker FOXP3 and the master negative regulator of immunity CTLA4.

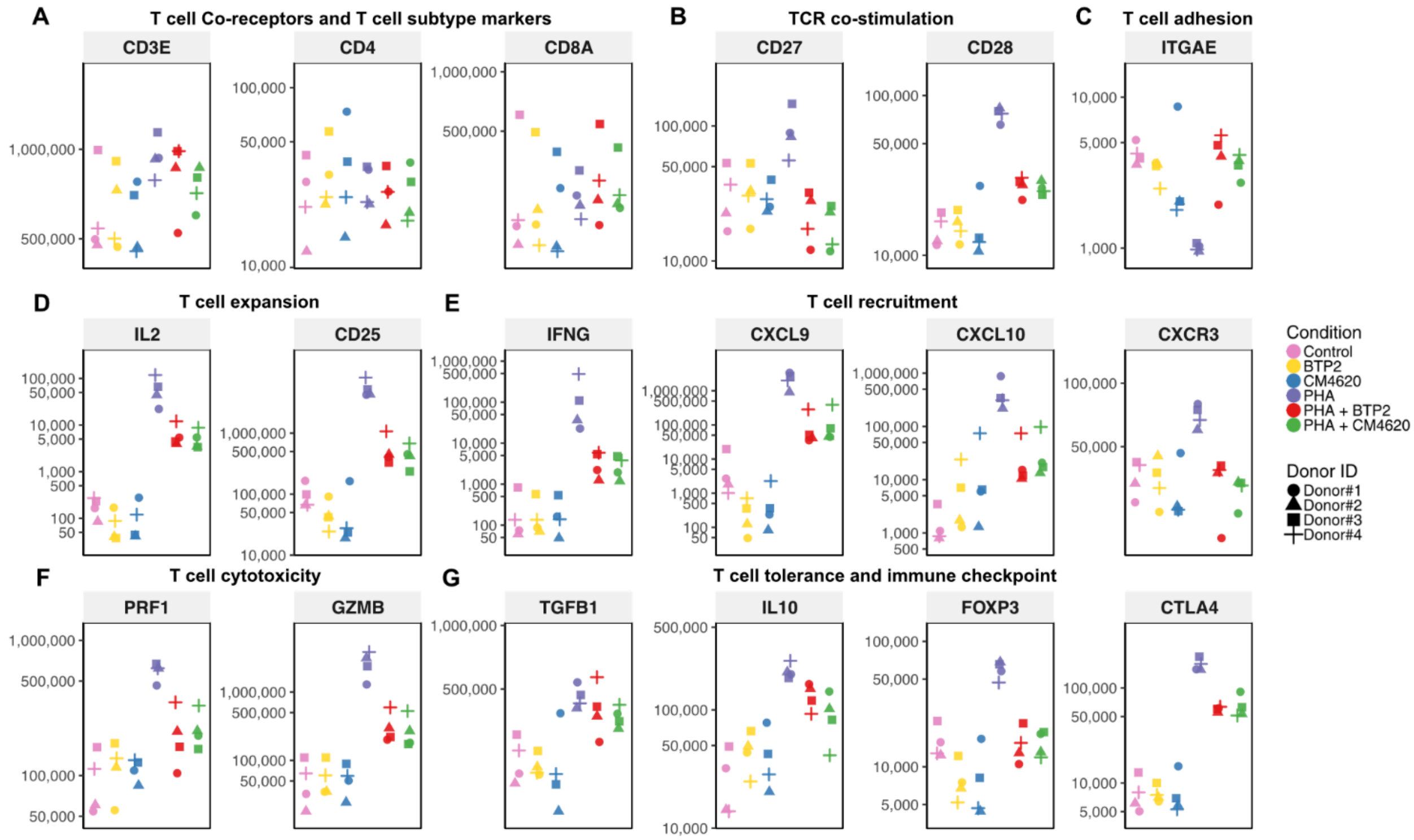

Supplementary Figure 4: RT-qPCR analysis of the effects of BTP2 and CM4620 on PHA-induced alterations in the expression of mRNAs in PBMCs. Jitter plots display the log-normalized absolute copy number of mRNA per microgram of total RNA across six experimental conditions. Target transcripts encode proteins involved in T cells expansion, recruitment, activation, effector function, and tolerance.

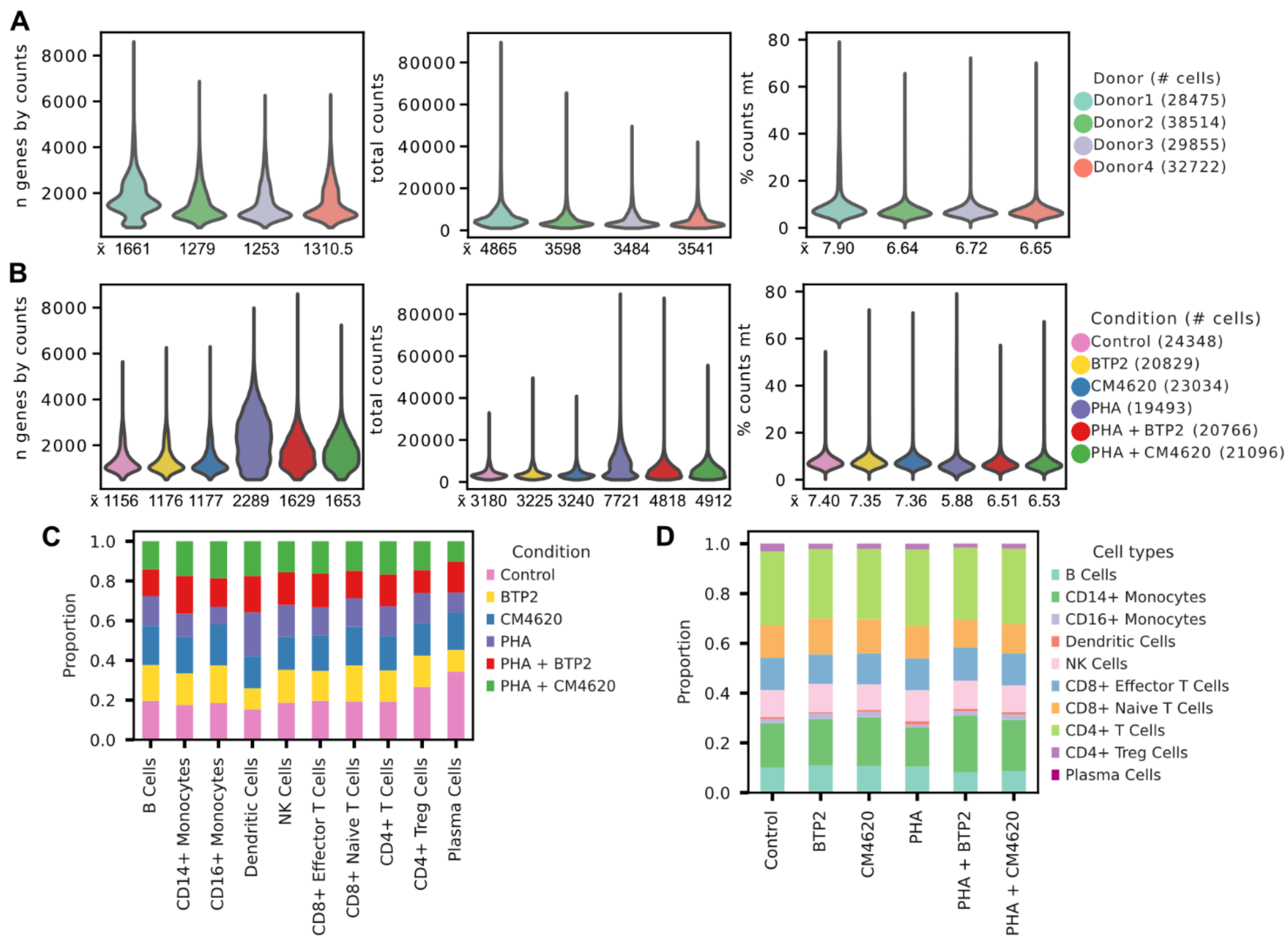

Supplementary Figure 5: Quality control metrics of scRNA-seq analysis. A-B) Violin plots showing the number of genes detected per cell (left), total transcript counts per cell (middle), and percentage of mitochondrial transcripts (right) in cells grouped by: **A)** Donor and **B)** Condition. Median per group indicated below each violin plot and number of cells next to the legend group. **C)** Fraction of cells attributed to various cell types split by condition. **D)** Fraction of cells attributed to various conditions split by cell type.

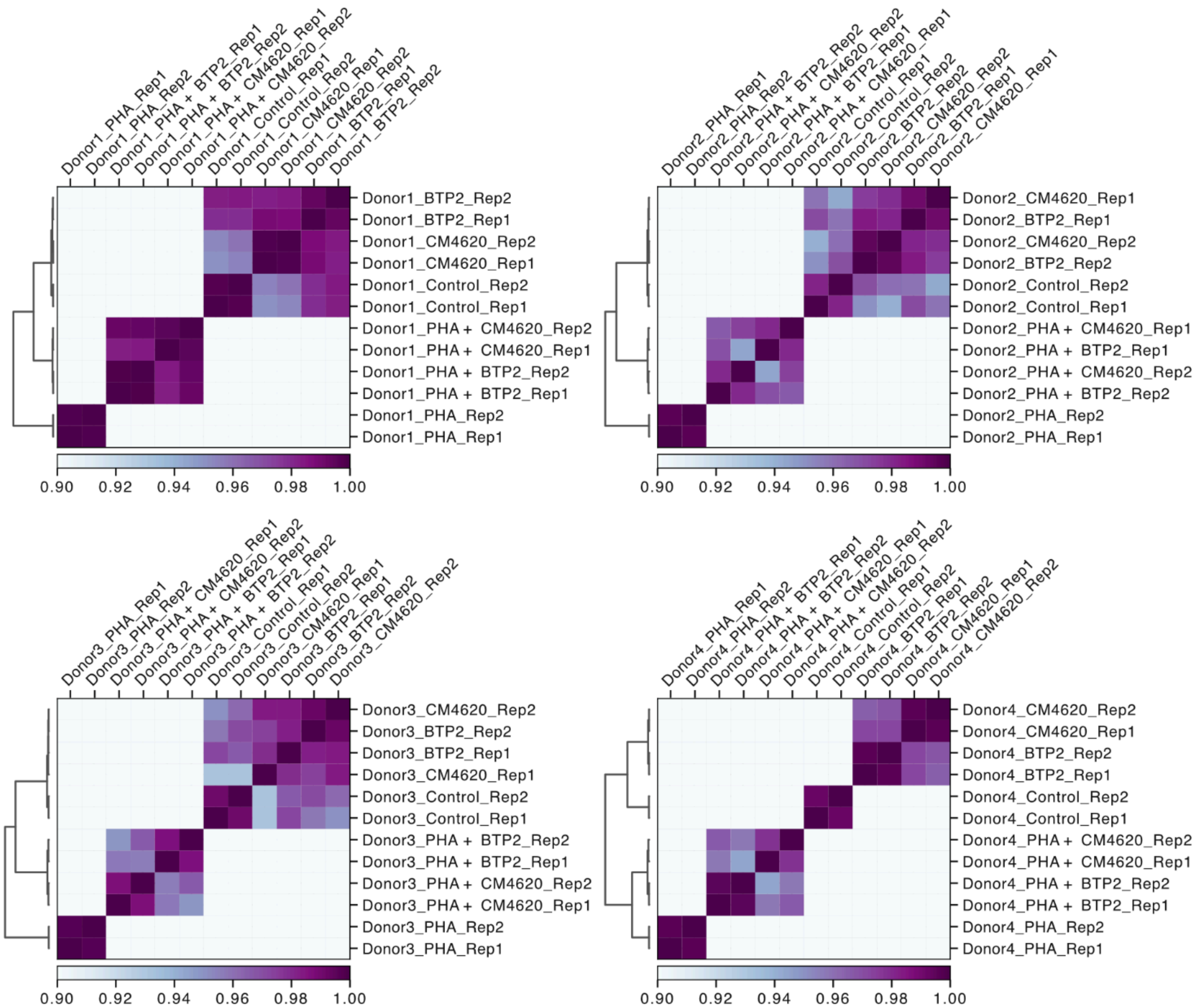

Supplementary Figure 6: **Heatmaps showing the pairwise Pearson correlation values for all scRNA-seq sample replicates across all treatment conditions within a donor**. Individual heat maps represent individual donors. Correlation analysis across samples within each donor revealed high correlations, typically ranging from 0.92 to 1.0, between technical replicates indicating strong reproducibility within each condition.

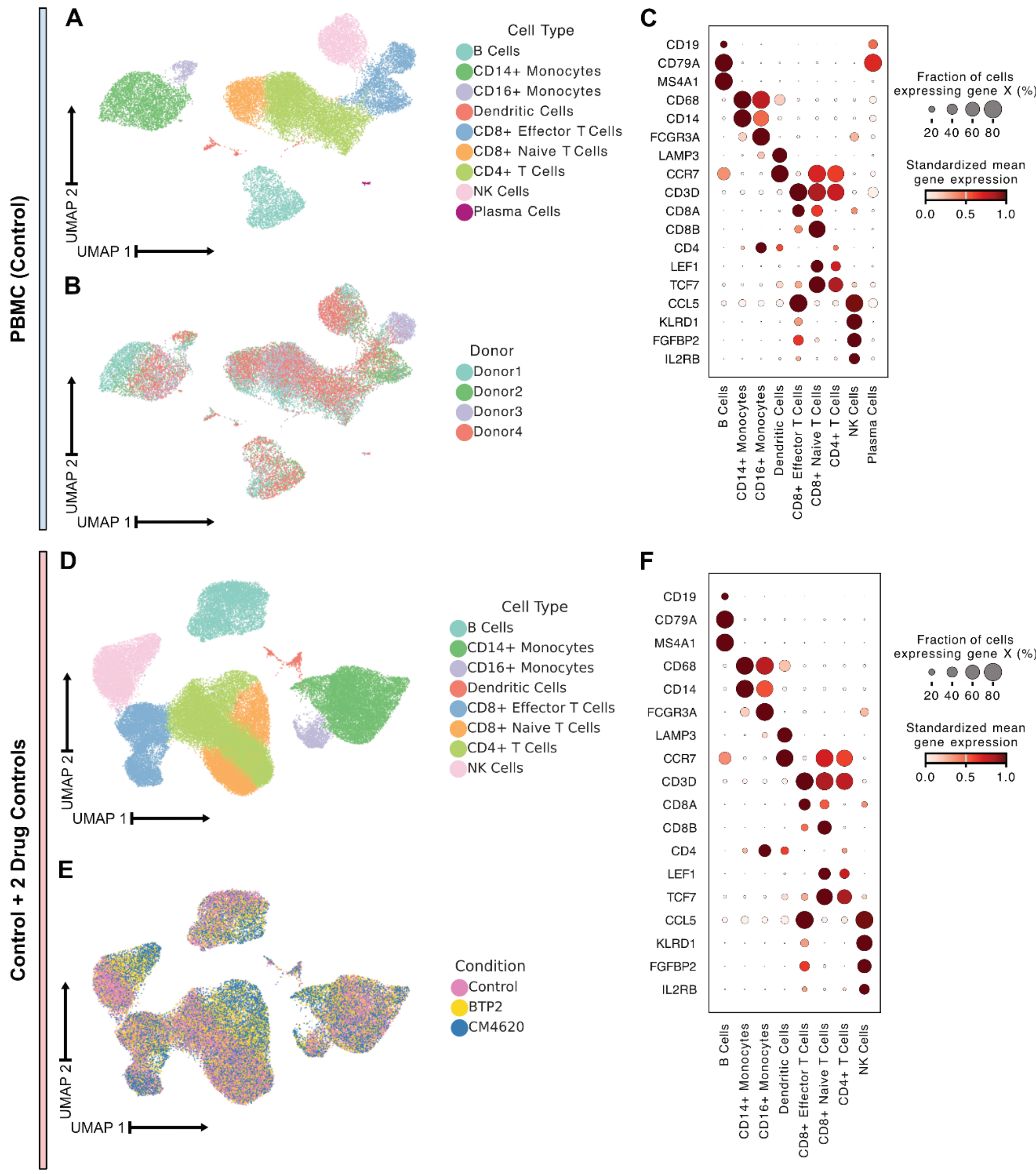

Supplementary Figure 7: scRNA-seq analysis of control PBMCs and of PBMCs treated with BTP2 or CM4620 combined. A) UMAP plot of single-cell transcriptomes from control PBMCs clustered by gene expression colored by cell type. **B)** UMAP plot of single-cell transcriptomes from control PBMCs clustered by gene expression colored by donor of origin. **C)** Dot plot showing the normalized gene expression for canonical cell type markers in individual cell types detected within control PBMCs. **D)** UMAP plot of single-cell transcriptomes from control PBMCs and unstimulated PBMCs treated with SOCE blockers clustered by gene expression colored by cell type. **E)** UMAP plot of single-cell transcriptomes from control PBMCs and unstimulated PBMCs treated with SOCE blockers clustered by gene expression colored by donor of origin. **F)** Dot plot showing normalized gene expression for canonical cell type markers in individual cell types detected within control PBMCs and unstimulated PBMCs treated with SOCE blockers.

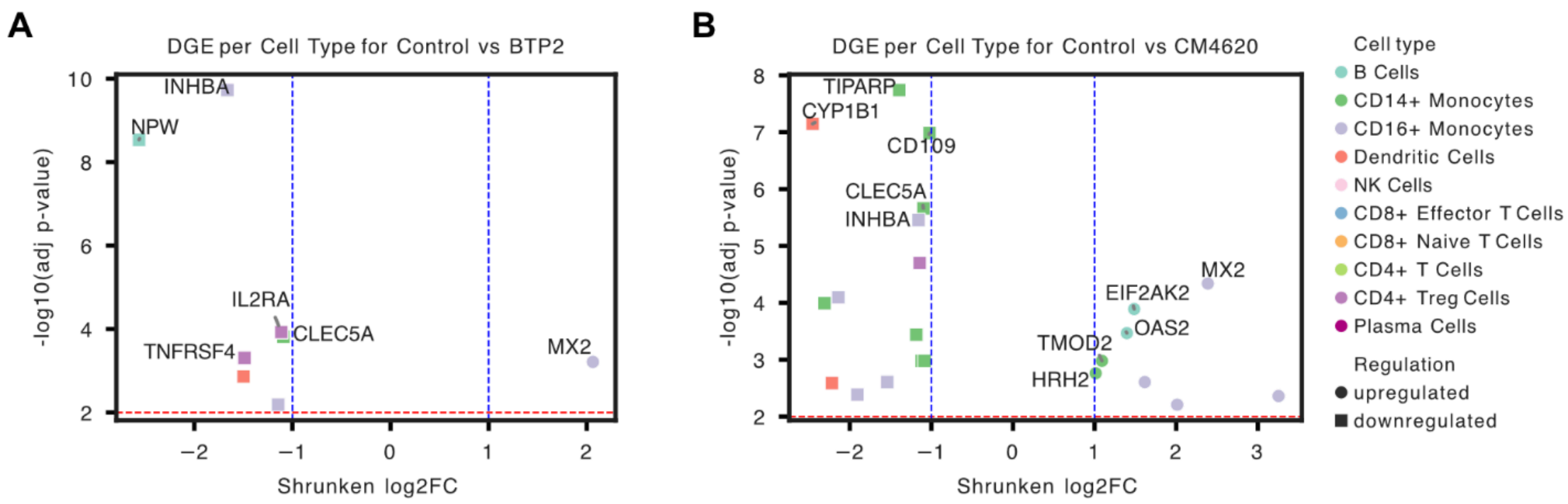

Supplementary Figure 8: DGE of control PBMCs vs. PBMCs+BTP2 and PBMCs vs. PBMC+CM4620 per cell type. A) Volcano plot showing DGEA results for PBMCs (Control) vs. BTP2 for each cell type. **B)** Volcano plot showing DGEA results for PBMCs (Control) vs. CM4620 for each cell type. **A-B)** Colors correspond to the cell type and the shape to the direction of regulation. The blue line defines the shrunken +/-1.0 $\log_2$ fold-change threshold and the red line is set at 0.01 adjusted p-value.

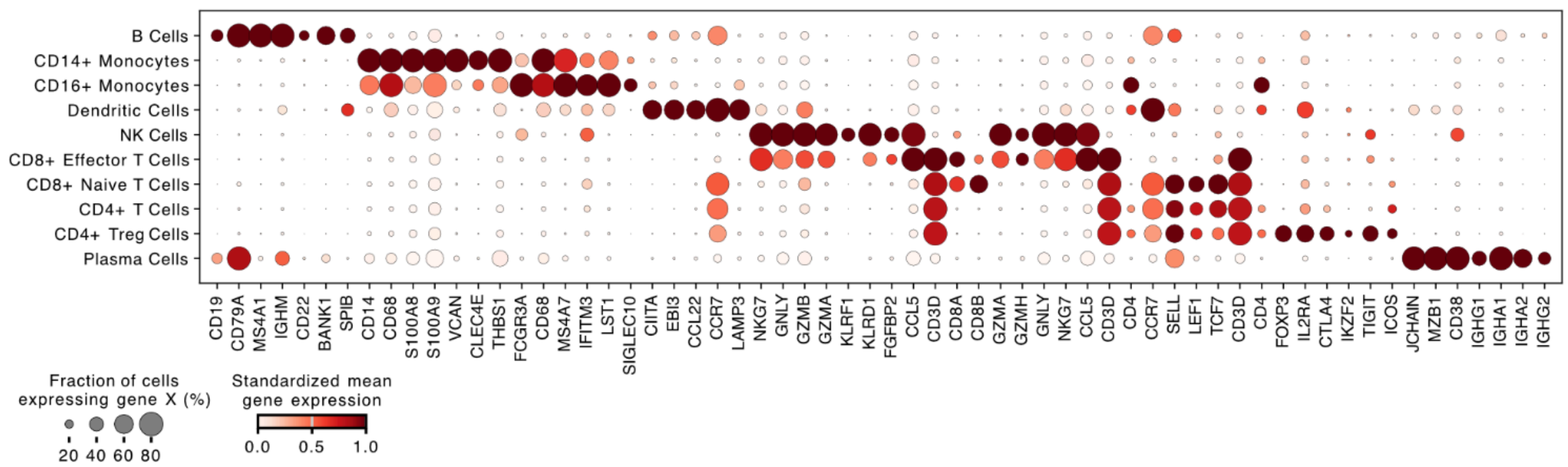

Supplementary Figure 9: Dot plot of extended marker gene list used for cell type annotations. Dot plot showing the normalized gene expression for canonical cell type markers in individual cell types detected within control PBMCs. Size of dots represents the fraction of cells that have a non-zero expression in a group. Color scale represents the standardized expression of a given gene for a group.

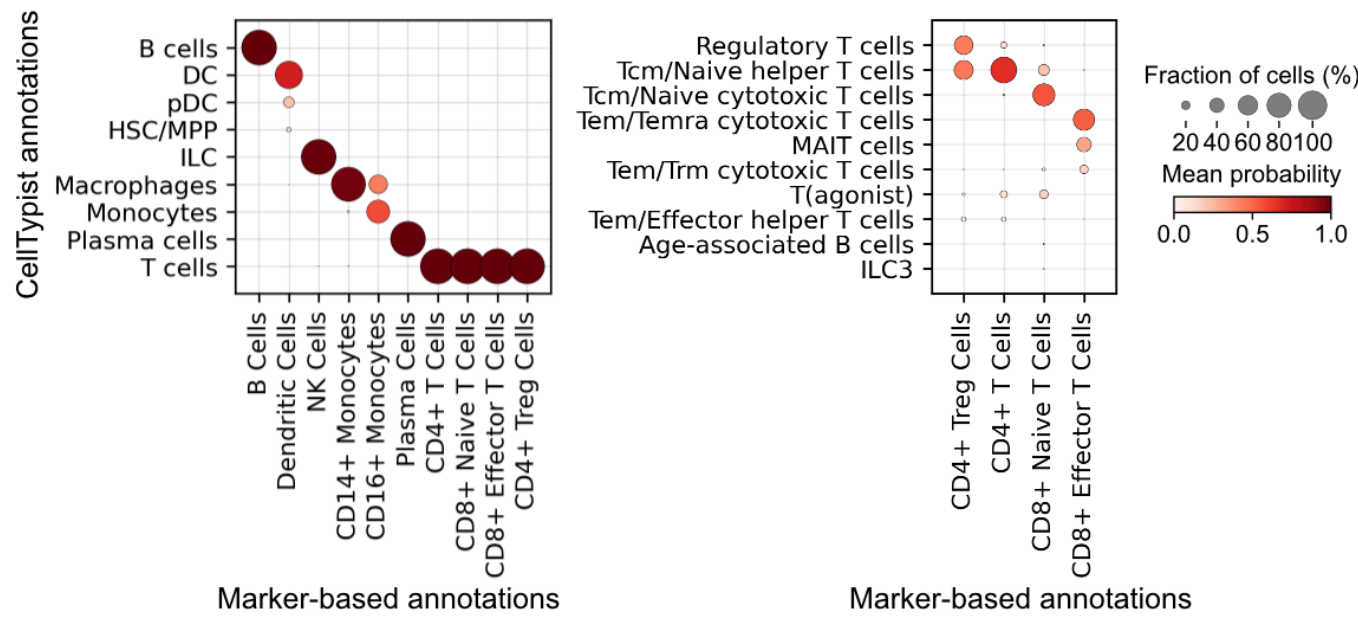

Supplementary Figure 10: Automated celltype annotation using CellTypist. Dot plots show the fraction of marker-based annotated cells assigned to each CellTypist label. Dot color represents the mean CellTypist prediction probability for each group. The left dotplot uses broad CellTypist immune cell annotations and the right uses refined CellTypist immune cell annotations.

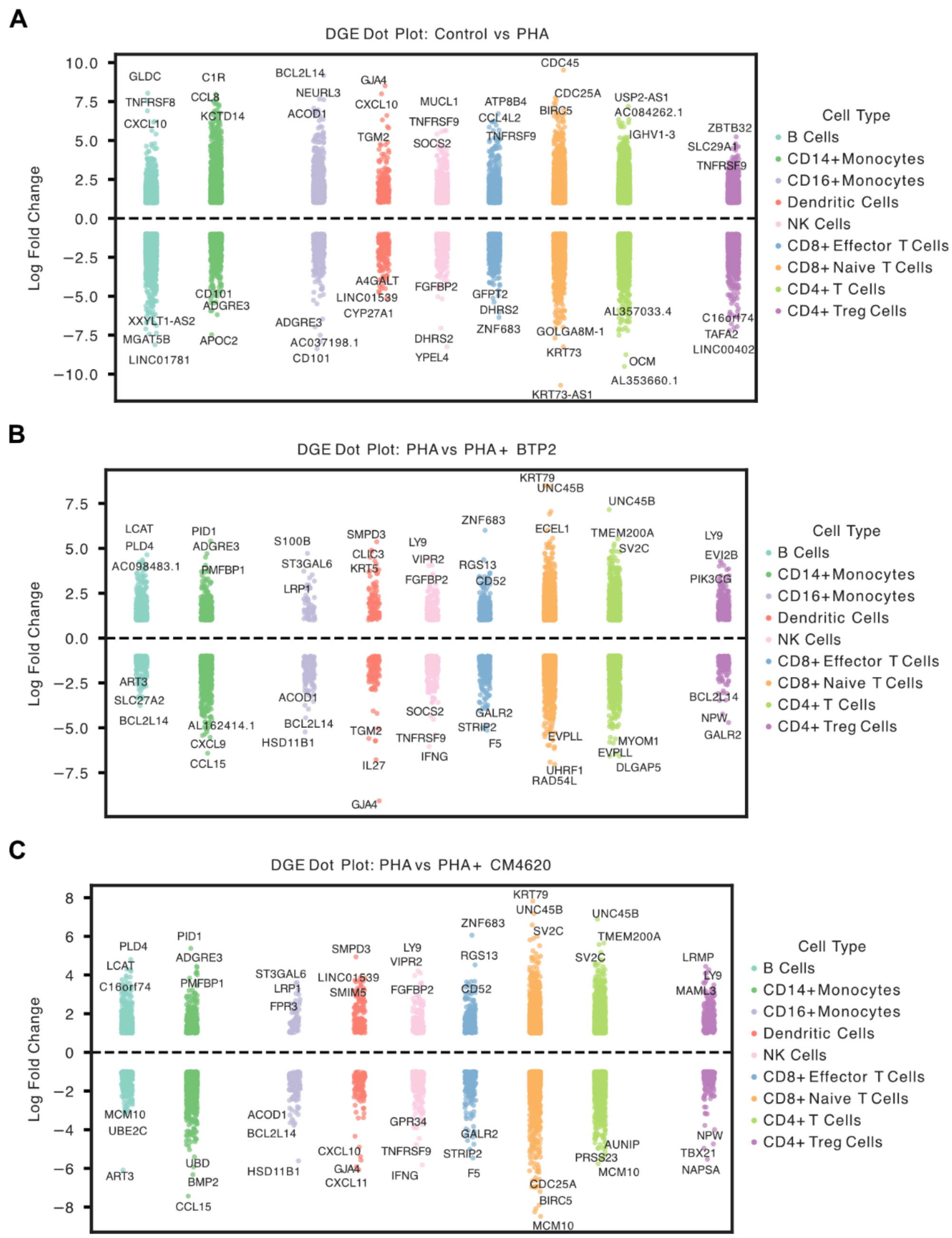

Supplementary Figure 11: Differential gene expression analysis between treatment conditions for all cell types except plasma cells. A) DGEA results for Control vs PHA. **B)** DGEA results for PHA vs PHA + BTP2. **C)** DGEA results for PHA vs PHA + CM4620. Only genes the top and bottom 3 genes that met the following thresholds were plotted: shrunken $\log_2$ fold-change > 1.0 or shrunken $\log_2$ fold-change < -1.0 and adjusted p-values < 0.01.

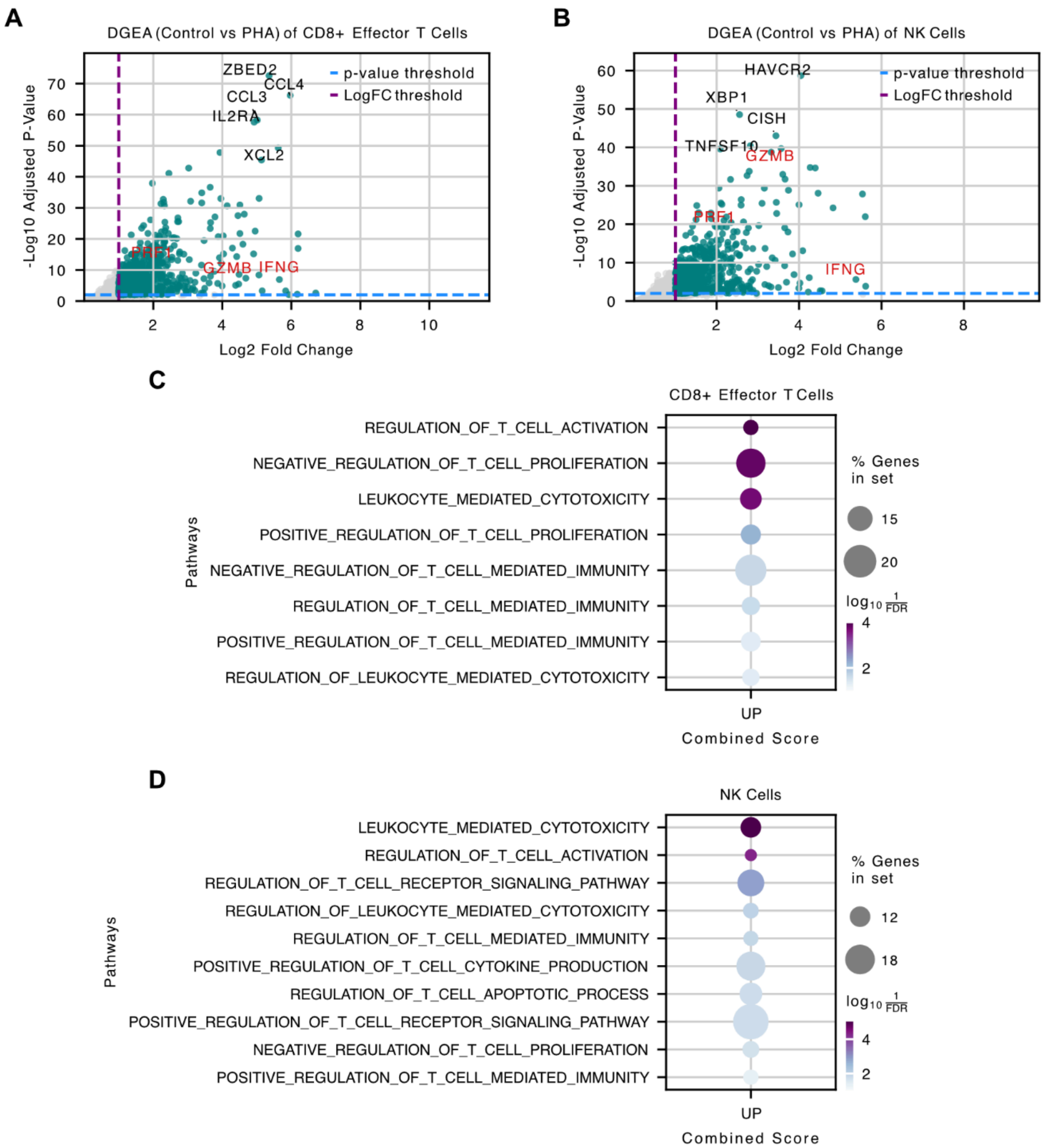

Supplementary Figure 12: Differential gene expression and gene set enrichment analysis between conditions: Control PBMCs vs. PBMCs + PHA. A) Volcano plot showing DGEA for CD8$^+$ Effector T cells in PHA-stimulated cells as compared to unstimulated controls. **B)** Volcano plot showing DGEA for NK cells in PHA-stimulated cells as compared to unstimulated controls. **A-B)** Pink dotted lines show the +1 log fold-change threshold, and the blue dotted lines show the adjusted p-value threshold of 0.01. Only the upregulated genes are plotted, and the top 5 upregulated gene names are shown in black while PRF1, GZMB, and IFNG in red. **C)** Dot plot showing top ontology terms enriched in DGEs for CD8$^+$ Effector T cells in PHA-stimulated PBMCs when compared to unstimulated controls. **D)** Dot plot showing top ontology terms enriched in DGEs for NK cells in PHA-stimulated PBMCs when compared to unstimulated controls. **C-D)** For gene set enrichment analysis only upregulated genes that met the following thresholds from the DGEA were used: shrunken $\log_2$ fold-change > 1.0 and adjusted p-values < 0.01.

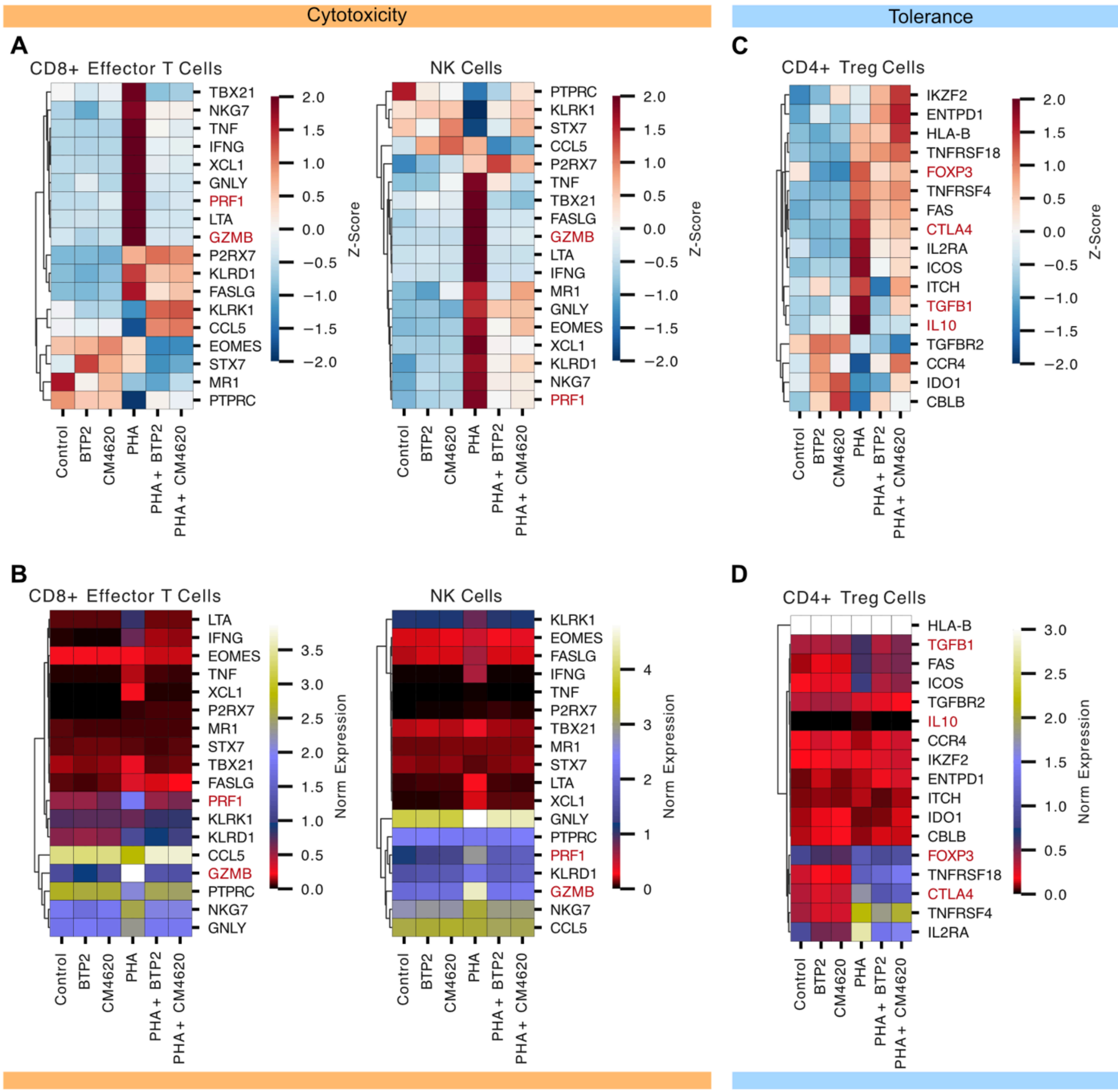

Supplementary Figure 13: **Gene expression of immune pathway related genes in T cells and NK cells. A)** Heatmaps showing z-scored normalized gene expressions of cytotoxic genes across different treatment conditions for CD8^{+} Effector T cells and NK cells. **B)** Heatmaps showing the normalized gene expression for cytotoxic genes across different treatment conditions for CD8^{+} Effector T cells and NK cells. **C)** Heatmaps showing z-scored normalized gene expressions of tolerance genes across different treatment conditions for CD4^{+} T-reg cells. **D)** Heatmaps showing the normalized gene expressions for tolerance genes across different treatment conditions for CD4^{+} T-reg cells. **A-D)** Red colored genes are the ones found in the RT-qPCR validation for cytotoxicity and tolerance.

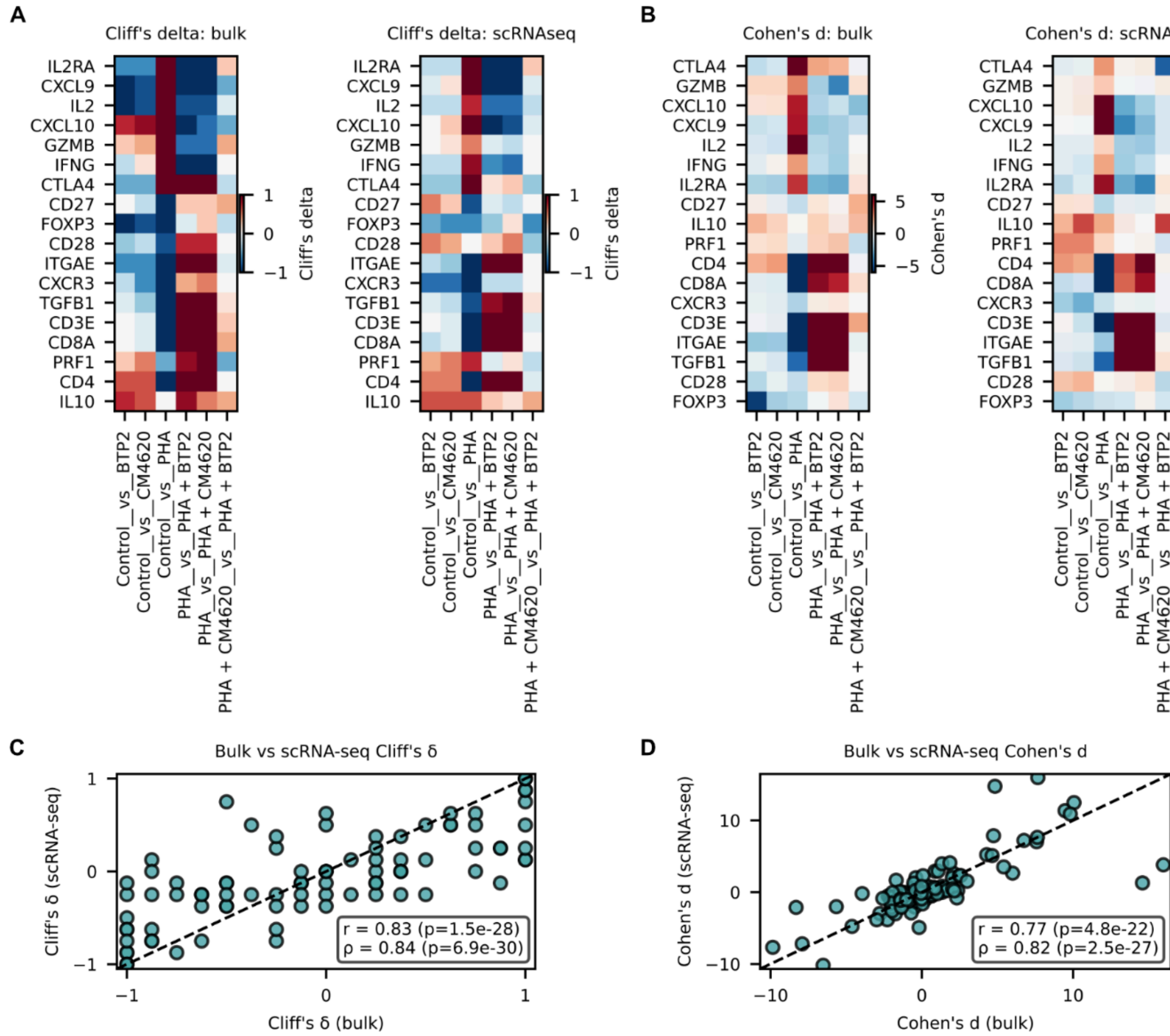

Supplementary Figure 14: Cross-dataset consistency of the effects of BTP2 and CM4620 on PHA-induced alterations in immune cell gene expression. Heatmaps showing **A)** Cliff's delta and **B)** Cohen's d effect size metrics for selected genes involved in T cell expansion, recruitment, activation, effector function, and tolerance across different condition comparisons and dataset modalities. Scatterplots comparing bulk and single-cell RNA-seq modalities for **C)** Cliff's delta and **D)** Cohen's d metrics. Pearson (r) and Spearman (ρ) correlation coefficients are indicated on each plot.

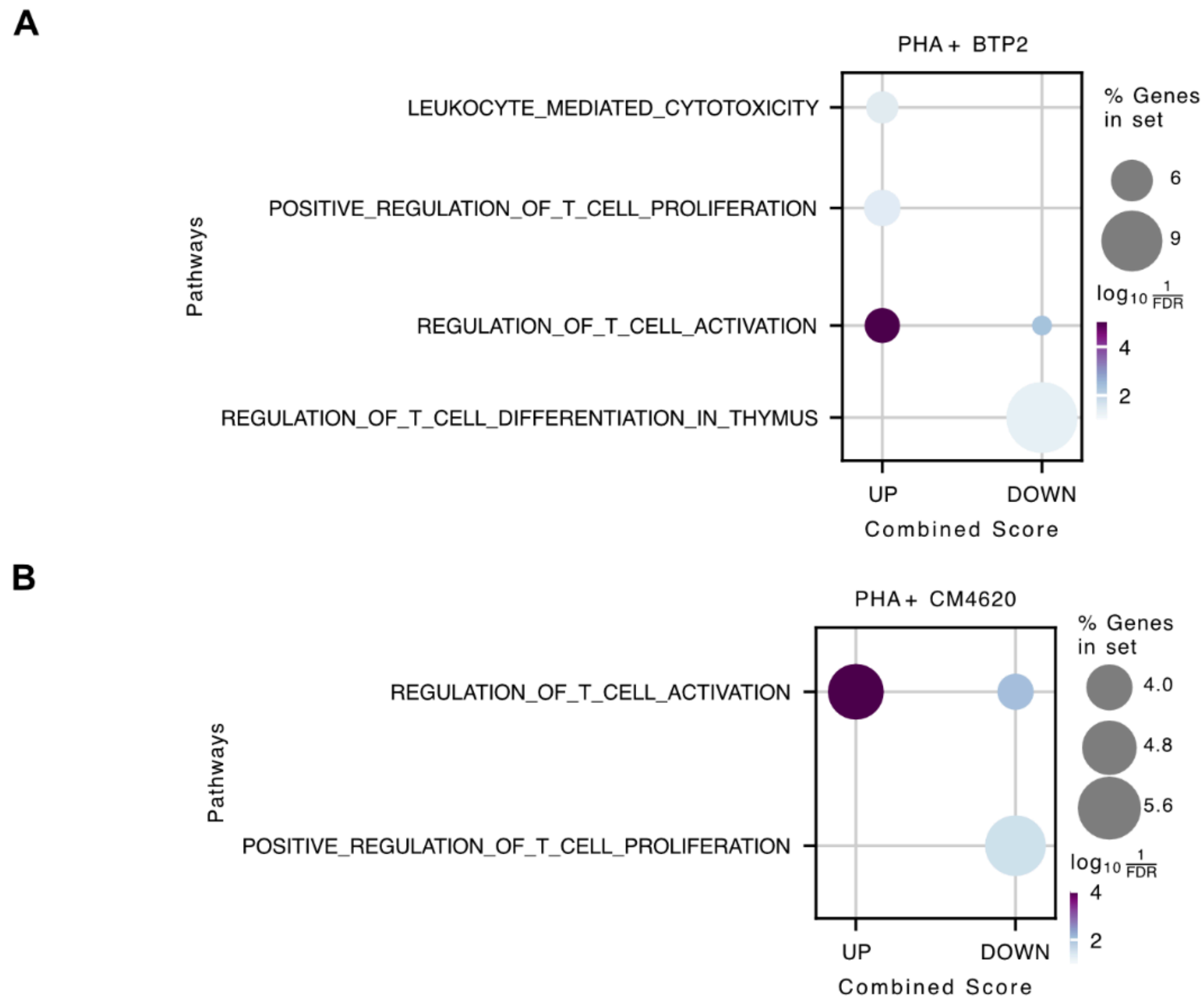

Supplementary Figure 15: **Gene set enrichment analysis for DGEA of CD4$^+$ T-reg cells between conditions A)** Dot plot showing ontology terms enriched in DGEA of Control vs. PHA + BTP2. **B)** Dot plot showing ontology terms enriched in Control vs. PHA + CM4620. For gene set enrichment analysis, only upregulated and downregulated genes that met the following thresholds from the DGEA were used: shrunken $\log_2$ fold-change > 1 for upregulated or shrunken $\log_2$ fold-change < -1 for downregulated and adjusted p-values < 0.01.
